# Targeting GABAergic Hypofunction Associated with Schizophrenia: Identification of α1β2γ2GABA‑A Receptor Ligands with Neuroprotective and Antipsychotic Properties

**DOI:** 10.1021/acschemneuro.5c00098

**Published:** 2025-06-06

**Authors:** Barbara Mordyl, Katarzyna Szafrańska, Joanna Sniecikowska, Jakub Jonczyk, Bartłomiej Bieńko, Maria Mateos-Jimenez, Bartosz Wojdyła, Beata Gryzło, Monika Głuch-Lutwin, Agata Siwek, Tadeusz Karcz, Karolina Słoczyńska, Elżbieta Pękala, Alicja Zakrzewska-Sito, Pawel Mierzejewski, Marcin Kołaczkowski, Monika Marcinkowska

**Affiliations:** † Faculty of Pharmacy, 49573 Jagiellonian University Medical College, 9 Medyczna Street, Krakow 30-688, Poland; ‡ Doctoral School of Medical and Health Sciences, Jagiellonian University Medical College, 16 św. Łazarza Street, Krakow 31-530, Poland; § 49794 Institute of Psychiatry and Neurology, 9 Sobieskiego Street, Warsaw 02-957, Poland

**Keywords:** GABA-A receptors, schizophrenia, psychosis, zolpidem, imidazo[1,2-*a*]-pyridines, nigrostriatal pathway

## Abstract

Clinical evidence has demonstrated significant hypofunction of GABAergic neurotransmission in patients with schizophrenia, likely contributing to the onset of psychotic symptoms. These symptoms can be alleviated by α1β2γ2GABA-A receptor ligands, which have previously shown antipsychotic activity. Building on this foundation, we synthesized and characterized various derivatives of 2-phenylimidazo­[1,2-*a*]-pyridine containing cyclic amine moieties at the amide backbone to identify potent ligands and expand the chemical space of α1β2γ2GABA-A receptor ligands. The synthesized compounds exhibited *K*_i_ values ranging from 25.0 to 7822.5 nM and positive allosteric properties at α1β2γ2GABA-A receptors. Selected compounds exhibited promising cellular permeability properties, high metabolic stability, and neuroprotective activity. A representative derivative of this series elicited antipsychotic-like properties, reversing amphetamine- and MK-801-induced hyperlocomotion without inducing sedative effects. Our findings indicate that α1β2γ2GABA-A ligands represent a promising strategy for the identification of potential antipsychotic agents with an original mechanism of action.

## Graphical abstract

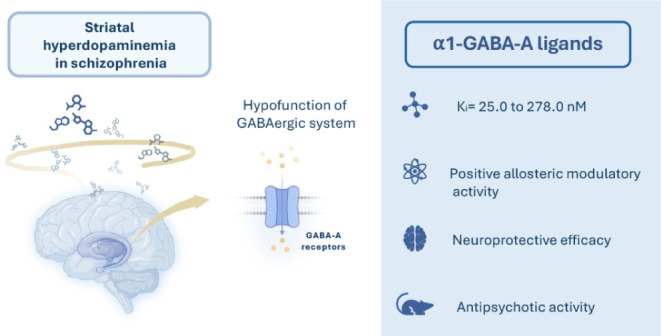

## Introduction

Schizophrenia continues to impose a significant socio-economic burden, with treatments still largely relying on scientific principles established 70 years ago.^1,2^ Although classical antipsychotic drugs derived from these findings have revolutionized the treatment of schizophrenia, they have shown limited effectiveness and are associated with significant side effects. Approximately 30% of patients with schizophrenia experience inadequate therapeutic response to current dopamine-blocking antipsychotics.^3,4^ This highlights the need to develop alternative approaches for controlling psychosis that do not rely on D_2_ dopamine receptor modulation.

Schizophrenia is a multifaceted disorder thought to arise from the complex interplay of multiple neurotransmitter systems.^5,6^ GABAergic dysfunction in schizophrenia has long been proposed and has ultimately been confirmed by both postmortem and clinical studies. One of the most replicated findings in postmortem analyses of brains from individuals with schizophrenia is a reduction in GAD67, the primary enzyme responsible for GABA synthesis in the brain.^7^ Further studies in patients with schizophrenia have identified alterations in other components of GABAergic neurotransmission in different brain regions. A positron emission tomography (PET) study using [^11^C]­flumazenil identified changes in GABA-A receptor binding in antipsychotic-naïve patients with psychosis,^8^ which was further confirmed by quantitative receptor autoradiography of postmortem tissue.^9^ These collective findings have given rise to the so-called GABAergic hypothesis of schizophrenia, suggesting that GABAergic dysfunction may be one of the potential root causes of the disorder.^10–12^

Importantly, the dysfunction of the GABAergic neurotransmitter system has been observed across multiple brain regions in schizophrenia patients, including the cortex, hippocampus, substantia nigra,^13–15^ and dorsal striatum.^15,16^ Post-mortem studies and molecular imaging analyses have revealed reductions in GABAergic markers in the substantia nigra of patients with schizophrenia, including decreases in the density of GABA synapses, reduced enzyme levels (e.g., GAD67), and substantial reductions in mRNA expression of the α1GABA-A receptor subunit (GABRA1 gene).^16–18^ It is important to note that GABAergic inhibitory neurons constitute the majority of inputs to dopaminergic neurons in the substantia nigra, playing a crucial role in regulating dopamine neuron activity.^19,20^ The loss of GABAergic inhibitory control of dopamine neurons in the striatum may contribute to dopamine and glutamate dysregulation, resulting in nigrostriatal dopaminergic hyperactivity, a phenomenon thought to play a role in psychosis associated with schizophrenia (Figure 1).^15,16,21,22^ Although psychosis may arise from diverse pathophysiological origins, GABAergic hypofunction in the nigrostriatal pathways—resulting in inadequate regulation of dopamine dysregulation and subsequent dopaminergic hyperactivity—is one of the proposed mechanisms underlying psychosis.^15–17,22^

**Figure 1:**
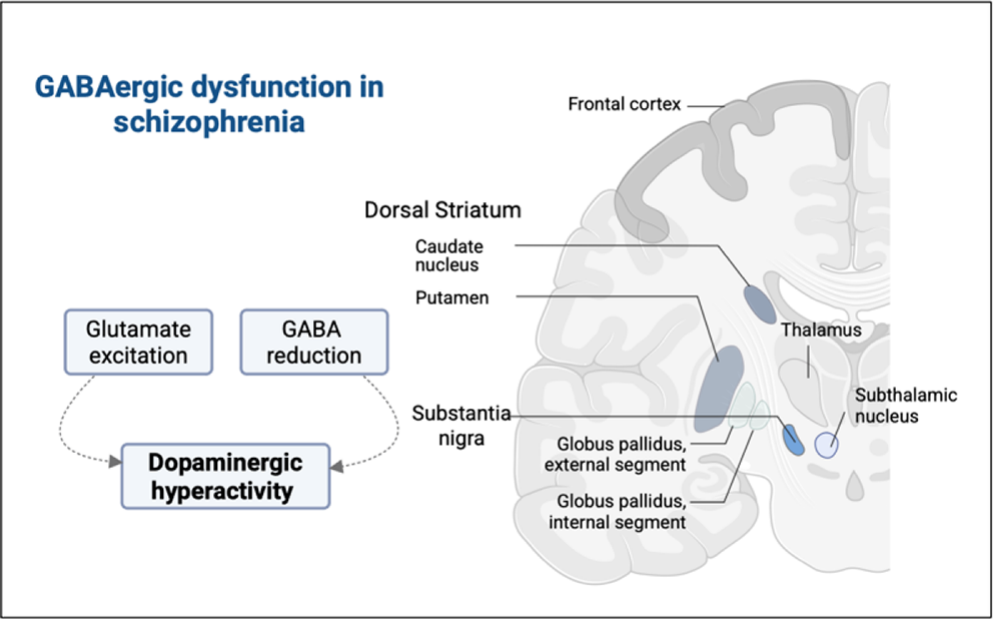
Schematic representation of GABAergic dysfunction in schizophrenia. Reduced GABAergic inhibition, along with excessive glutamatergic excitation, may lead to dopaminergic hyperactivity.

Nevertheless, clinical evidence of GABAergic dysfunction observed at multiple levels in patients with schizophrenia has prompted clinical trials evaluating the efficacy of benzodiazepines in treating psychotic symptoms associated with the disorder.^23^ However, these trials have yielded inconsistent results, indicating that the benefits are short-term and support their use as adjunctive therapy rather than monotherapy for the treatment of schizophrenia-like psychoses. This aligns with our animal studies, where diazepam (a nonselective GABA-A modulator) failed to demonstrate specific antipsychotic efficacy and primarily induced myorelaxant and ataxic effects in rodents.^24^ In striking contrast, we observed that more selective GABA-A PAMs, preferential for the α1 receptor subtype, such as zolpidem^24^ and its structural analogues,^25,26^ elicited a specific antipsychotic effect at subsedative doses.

These specific antipsychotic properties of zolpidem may stem from its ability to control nigrostriatal dopaminergic signaling.^16^ It is noteworthy that the α1GABA-A receptor subpopulation is highly expressed in the basal ganglia,^27^ with autoradiographic studies demonstrating a high binding density for zolpidem in the rat globus pallidus and substantia nigra pars reticulata (SNr).^28–30^ Numerous clinical trials have reported unusual neurological effects of zolpidem in patients with various neurological disorders, attributed to its selective modulation of α1GABA-A receptor subtypes in the basal ganglia.^31,32^ Interestingly, among the various documented cases, there was a report of a patients with schizophrenia in whom the antipsychotic effect of zolpidem was observed.^33,34^ Despite its promising pharmacological activity, zolpidem presents notable limitations in emerging therapeutic applications such as the treatment of psychosis, primarily due to its specific pharmacokinetic characteristics. Originally developed as a short-acting hypnotic agent for insomnia, zolpidem undergoes extensive hepatic metabolism, resulting in a short plasma half-life (t_0,5_ = 30.6 min in rats,^35^ ≈2 h in humans^36^). While this rapid elimination is beneficial for sleep induction, it poses a significant challenge in neuropsychiatric settings that require sustained receptor engagement. Studies consistently indicate that the therapeutic benefits of zolpidem in patients presenting with a variety of neurological disorders are short-lived, typically resolving within few hours.^37^

To overcome these limitations, the development of novel analogues that retain high affinity for the α1β2γ2GABA-A receptor while exhibiting extended duration of action and improved pharmacokinetics may help address the shortcomings of zolpidem in the identification of antipsychotic agents based on a novel, nondopaminergic mechanism of action. Herein we report a new series of 2-(4-fluorophenyl)-6-methylimidazo­[1,2-*a*]­pyridine derivatives bearing amide-linked cyclic amines, designed as positive allosteric modulators (PAMs) of the α1β2γ2 GABA-A receptor with improved pharmacokinetic profile. The compounds from this series were evaluated for their binding affinity and PAM efficacy at α1β2γ2 GABA-A receptor and were subjected to early ADME-Tox profiling. Selected compounds were assessed for their neuroprotective potential in a 6-hydroxydopamine-induced neurotoxicity assay, given prior evidence that GABA-A receptor ligands exert neuroprotective effects.^38,39^ Our lead compound, **17**, identified through a cascade of *in vitro* assays, retains high α1β2γ2GABA-A receptor affinity and efficacy while exhibiting a longer half-life and favorable CNS penetration. As a result, it produced robust antipsychotic-like effects in rodent models without the pronounced acute sedation that accompanies higher doses of zolpidem. Our work expands the chemical space of α1β2γ2GABA-A receptor ligands with favorable pharmacokinetic profiles and contributes to the identification of novel nondopaminergic antipsychotic candidates acting through modulation of GABAergic neurotransmission. Notably, α1β2γ2 GABA-A ligands not only alleviate psychotic symptoms but have also demonstrated neuroprotective effects, further underscoring their potential as therapeutic agents for schizophrenia, particularly in light of the growing interest in neuroprotection as a novel treatment strategy.

## Results and Discussion

### Design and Synthesis of α1β2γ2 GABA-A Receptor PAMs

Previously, we identified a series of α1β2γ2 GABA-A receptor PAMs that demonstrated antipsychotic activity in animal studies and improved metabolic stability compared to zolpidem. Notably, zolpidem, a 6-methyl-2-(*p*-tolyl)­imidazo­[1,2-*a*]­pyridine derivative, contains two methyl groups (at the 6 and 4’ positions) that act as metabolic hotspots, with the 4’-methyl group being particularly prone to metabolic degradation^36,40^ (Figure 2). This metabolic vulnerability contributes to zolpidem’s short half-life, which is approximately 30.6 min in rats^35^ and 2–3 h in human plasma.^36^ Through a series of modifications, we demonstrated that introducing a single-site fluorination at the position most susceptible to metabolic degradation (4’) yields the 2-(4-fluorophenyl)-6-methylimidazo­[1,2-*a*]­pyridine scaffold, which exhibits improved metabolic stability compared to its methyl-substituted counterparts.^25,26,41^ At the same time, derivatives of this fluorinated 2-phenylimidazo­[1,2-*a*]-pyridine scaffold were devoid of the hepatotoxicity that may occur when both methyl groups are replaced with halogen atoms.^42^ Structure–activity relationships (SAR) around this scaffold revealed that introducing specific modifications at the amide terminal can enhance activity toward α1β2γ2GABA-A receptor ligands, with a trend toward cyclic amine derivatives. Relying on these findings, we hypothesized that investigating various cyclic amine derivatives around the amide tail of fluorinated 2-phenylimidazo­[1,2-*a*]-pyridines would yield α1β2γ2GABA-A receptor ligands with high affinity and positive allosteric modulation efficacy (Figure 2). The choice of specific substituents for the amide end was based on synthetic feasibility, alignment with Lipinski’s Rule of 5 and CNS MPO. The library of fluorinated 2-phenylimidazo­[1,2-*a*]-pyridine derivatives, containing amides of cyclic amines, was synthesized according to a four-step protocol (Scheme 1).

**Figure 2:**
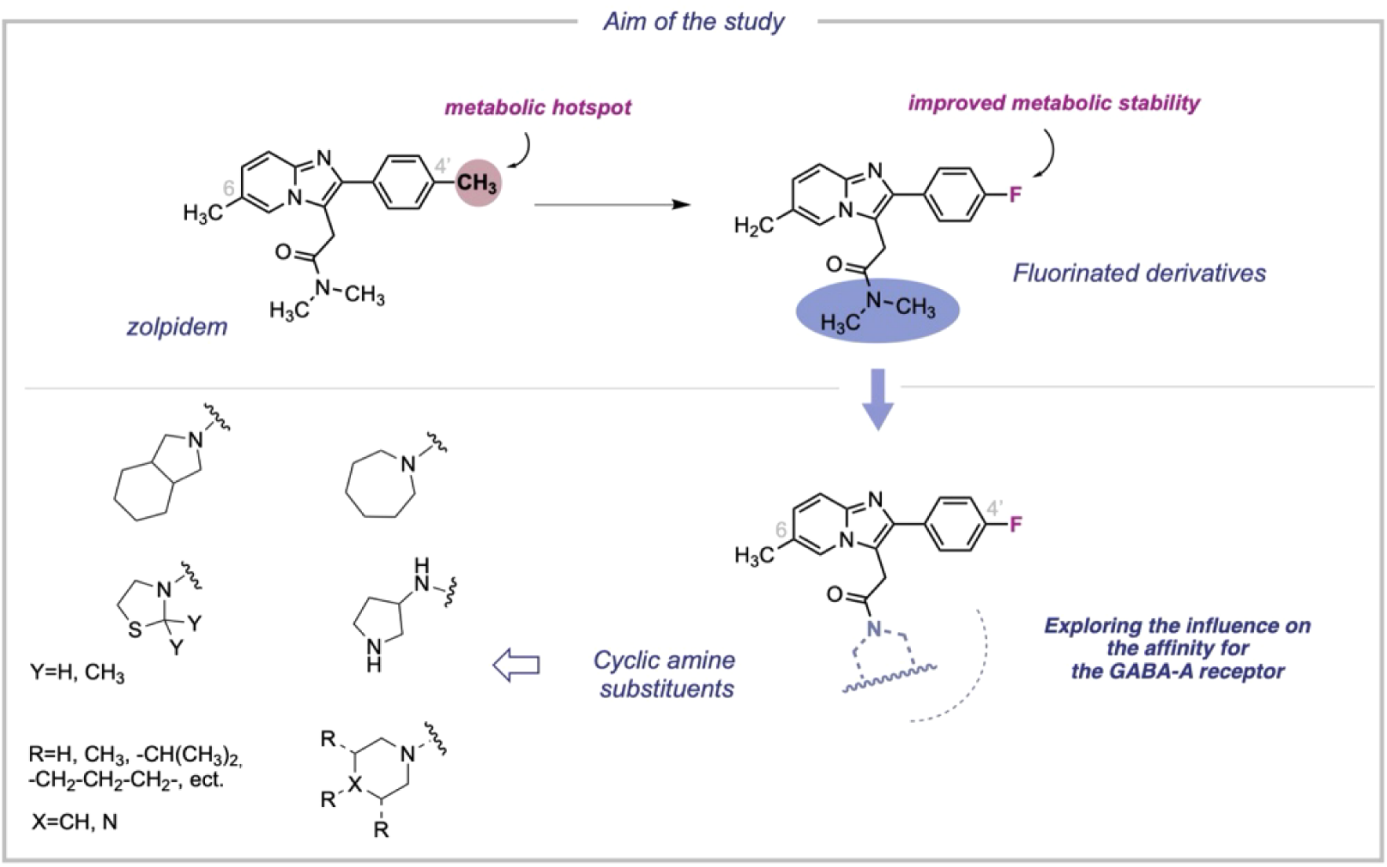
Design of novel series of 2-phenylimidazo­[1,2-*a*]-pyridine derivatives.

**Scheme 1:**
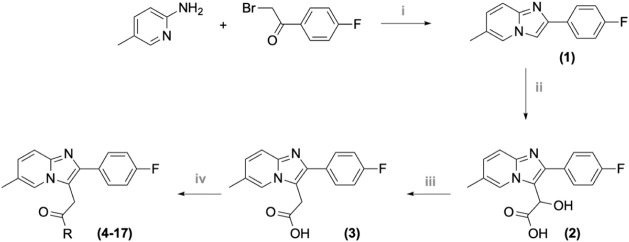
Synthesis of Fluorinated 2-Phenylimidazo­[1,2-*a*]-Pyridine Derivatives (**4–17**)­^a^

a (i) Sodium bicarbonate, toluene, 100 °C, microwave, 1 h (90%); (ii) glyoxylic acid, DCM, 45 °C, 16 h (90%); (iii) Pd/C, HCOOH, 100 °C, 16 h (93%); (iv) cyclic amine, CDI, THF, 40 °C, 12 h (25–65%).

The synthesis began with the condensation under microwave irradiation of two building blocks: 2-bromo-1-(4-fluorophenyl)­ethan-1-one and 5-methylpyridin-2-amine, yielding 2-(4-fluorophenyl)-6-methylimidazo­[1,2-*a*]­pyridine (**1**) (Scheme 1).^43^ The 2-phenylimidazo­[1,2-*a*]-pyridine core (**1**) was reacted with glyoxylic acid to yield the α-hydroxyacetic acid derivative (**2**), which was subsequently subjected to dehydroxylation to produce the key building block (**3**). The final molecules (**4**–**17**) were prepared by reacting the key acid (**3**) with the appropriate cyclic amine in the presence of CDI.

### Structure–Activity Relationships

All the synthesized compounds (**4–17**) were subjected to a radioligand binding study using [^3^H]­flunitrazepam to establish the *K*_i_ value (Table 1). The affinity for α1β2γ2GABA-A receptor varied, with observed *K*_i_ values ranging from 29.0 to 7822.5 nM. The highest affinity was observed for the piperazine derivative (**5**) (*K*_i_ value of 29.0 ± 3.8 nM). Introduction of the 2,6-dimethylpiperazine (**6**) resulted in only a slight drop in affinity compared to the unsubstituted piperazine (**5**). Introduction of substituents at the 4-N position of the piperazine ring resulted in a marked reduction in affinity for the GABA-A receptor. For example, incorporation of a methyl group to yield 4-methylpiperazine (**4**) led to a 10-fold decrease in affinity (*K*_i_ = 278.0 ± 43.0 nM). Moreover, the presence of bulkier substituents such as isopropyl (**8**), cyclopropyl (**9**), isobutyl (**10**) or *tert*-butyl (**11**) caused a pronounced drop in affinity (*K*_i_ from 1336.0 ± 46.0 nM to 7822.5 ± 1720.5 nM). Among the remaining analogues, the 4-methylpiperidine (**7**) derivative also exhibited lower affinity compared to the piperazine scaffold, with a *K*_i_ value of 204 nM. Cyclic amines such as octahydro-1*H*-isoindole (**12**) and azepane (**13**) resulted in reduced affinity, with *K*_i_ values of 517 nM and 453 nM, respectively. Five-membered ring derivatives were generally well tolerated, as exemplified by the thiazolidine analogue **15**, which displayed high affinity for the GABA-A receptor (*K*_i_ = 63.0 ± 8.2 nM). This activity was comparable to that of the pyrrolidine derivative (**17**, *K*_i_ = 54.0 ± 3.6 nM) obtained previously.^41^ In contrast, the 2,2-dimethylthiazolidine analogue (**14**) exhibited significantly reduced affinity, with a nearly 10-fold increase in *K*_i_ = 551 nM). Introduction of pyrrolidin-3-amine (**16**) resulted in a drop in affinity (*K*_i_ = 140.0 ± 5.5 nM) compared to the piperazine derivative (**5**).

**Table 1:**
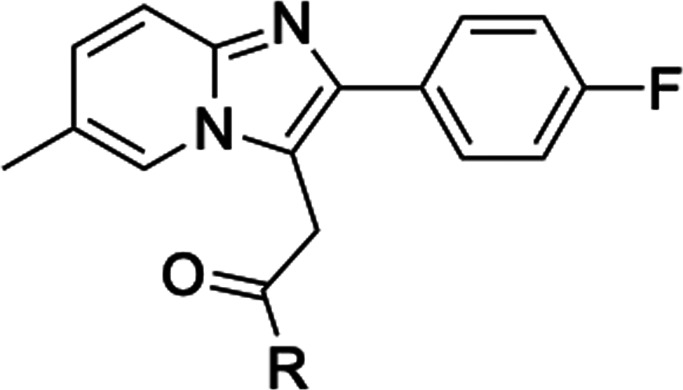

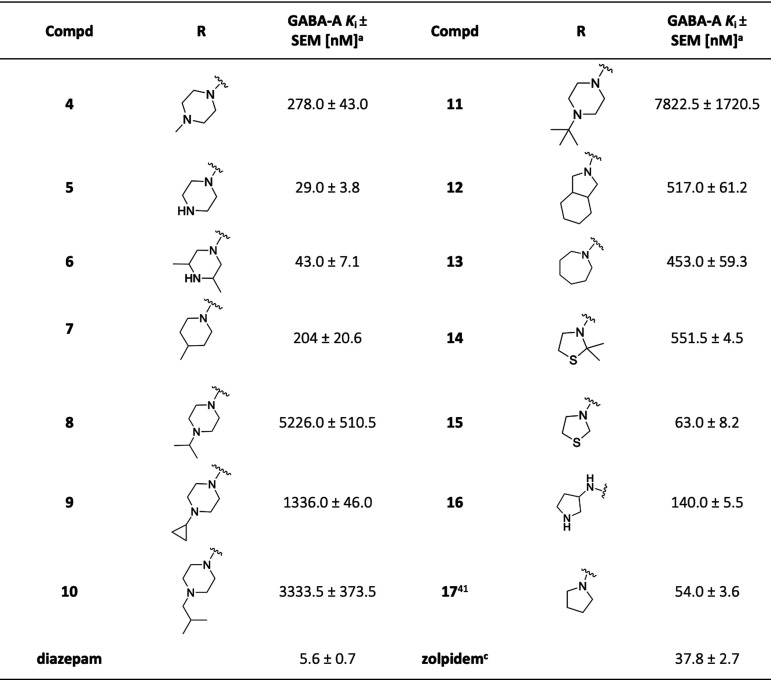
Binding Affinities (*K*_i_) at the GABA-A Receptor

a Data are represented as the mean *K*_i_ ± SEM of two-three independent experiments.

Based on the *K*_i_ data, compounds with *K*_i_ values below 200 nM (**5**, **6**, **15**, **16**, **17**) were selected for electrophysiological studies to assess their PAM efficacy at the α1β2γ2 GABA-A receptor (Table 2). The selected compounds potentiated GABA-induced Cl^–^ currents, confirming their allosteric modulatory properties. The piperazine analogue (**5**) showed the highest PAM efficacy among the novel series with EC_50_ = 543 ± 71 nM, while the 2,6-dimethylpiperazine analogue (**6**) displayed the highest maximal modulation of GABA-induced chloride currents (*E*_max_ = 2.78 ± 0.08, surpassing the FDA-approved drugs zolpidem and diazepam, Table 2). For the pyrrolidin-3-amine (**16**) derivative we observed lower efficacy in the recorded currents, with an EC_50_ = 2 490 ± 470 nM and an *E*_max_ value of 1.90 ± 0.11. The five-membered ring derivative containing a thiazolidine ring (**15**) displayed high PAM efficacy (EC_50_ = 743 ± 136 nM), comparable to the five-membered cyclic amine derivative, pyrrolidine (**17**) (EC_50_ = 910 ± 198 nM). The observed *E*_max_ value for compound **15** (*E*_max_ = 2.30) was similar to those observed for the reference compounds zolpidem and diazepam.

**Table 2:** PAM Efficacies at α1β2γ2 GABA-A receptor, and Permeability Values Measured in PAMPA Assay for Selected Compounds

Compd	EC_50_ ± SEM [nM]^a^	*E*_max_ ± SEM (fold increase of 10 μM GABA efficacy)	Permeability P_e_ ± SD [·10^–6^ cm/s]
**5**	543 ± 71	2.60 ± 0.12	3.35 ± 0.40
**6**	872 ± 77	2.78 ± 0.08	16.8 ± 3.5
**15**	743 ± 136	2.30 ± 0.57	7.89 ± 1.35
**16**	2 490 ± 470	1.90 ± 0.11	0.154 ± 0.005
**17**	910 ± 198	2.50 ± 0.42	29.7 ± 0.86 ^41^
**diazepam**	360 ± 55	2.30 ± 0.11	
**zolpidem** ^b^	195 ± 55	2.31 ± 0.12	21.6 ± 1.26
**sulpiride**			0.016 ± 0.003
**caffeine**			15.2 ± 3.0

a EC_50_ ± SEM values from electrophysiological experiments are given as mean of three independent experiments.

b PAMPA assay reported on zolpidem tartrate.^44^ Blank spaces—not tested.

Given that permeability is a fundamental prerequisite for achieving effective intracellular concentrations necessary for proper efficacy in biological systems, we measured the artificial permeability of our series using the parallel artificial membrane permeability (PAMPA) assay.^45^ The permeability of tested compounds was ranging from P_e_= 0.154 × 10^–6^ cm/s up to P_e_ = 16.8 × 10^–6^ cm/s. The highest permeability was observed for the 2,6-dimethylpiperazine (**6**) derivative (P_e_ = 16.8 ± 3.5 × 10^–6^ cm/s) which is slightly higher than the reference compound caffeine (P_e_ = 15.2 ± 3.0 × 10^–6^ cm/s), known to be highly permeable in this assay.^46^ The thiazolidine derivative (**15**) exhibited fair permeability (P_e_ = 7.89 ± 1.35 × 10^–6^ cm/s). The piperazine derivative (**5**) elicited relatively lower P_e_ values (P_e_ = 3.35 ± 0.40 × 10^–6^ cm/s). Nevertheless, for subsequent cellular studies, we selected compounds with P_e_ values > 4 × 10^–6^ cm/s (compounds **6**, **15** and **17**), as previous studies have indicated that compounds with P_e_ values less than 4.0 × 10^–6^ cm/s are generally poorly permeable through the blood-brain barrier.^45^

### Molecular Modeling

We used computer modeling techniques to assess how the proposed substituents influenced the interaction between the tested compounds and their primary target, the GABA-A receptor. For consistency with other experiments performed, we focused on the detailed analysis of the binding mode of compound **6**.

The docking studies demonstrated consistent binding of the tested compounds. When binding to the area between the α1 and γ2 subunits of the extracellular domain (ECD), the compounds adopt conformations similar to zolpidem. In Figure 3 one can observe how the 2-phenylimidazo­[1,2-*a*]­pyridine fragment fits into the hydrophobic pocket built by, among others, aromatic amino acids Phe-100, Tyr-160, Tyr-210 or Phe-77. In addition, the position adjacent to Phe-77 permits the establishment of an aromatic π-π stacking interaction.

**Figure 3:**
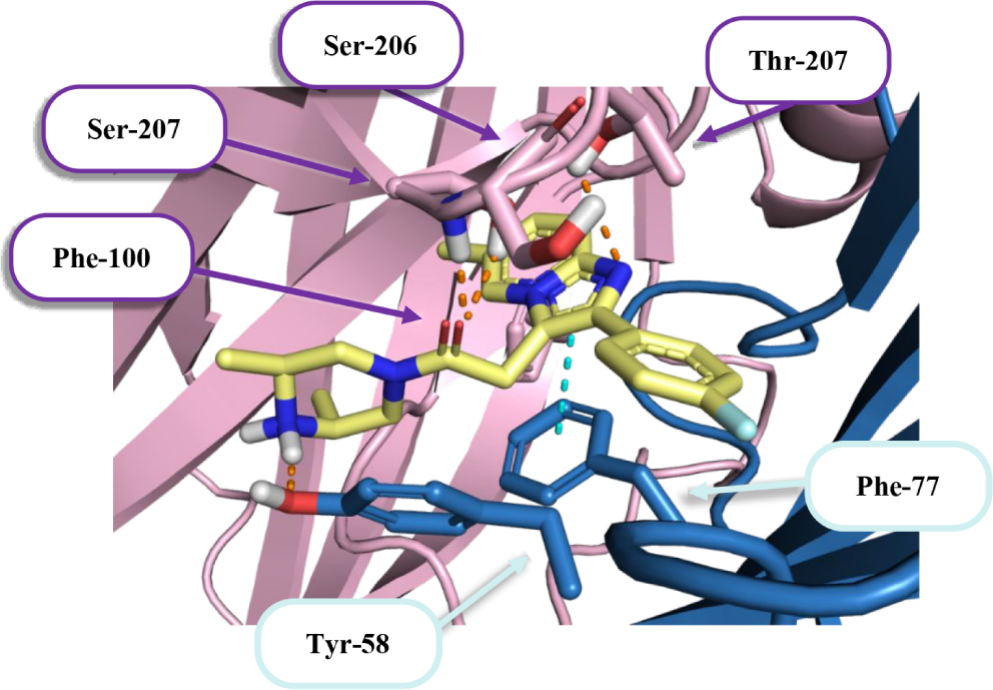
Predicted interaction modes of compound **6** with the GABA-A receptor (PDB: 8DD2) at the benzodiazepine binding site. The α1-subunit is marked in pink and the γ2-subunit in dark blue, with interacting amino acids represented by sticks. Orange dots indicate hydrogen bonds and cyan represents aromatic interactions, including both CH−π and π–π.

One of the key elements of the interaction with ECD are hydrogen bonds between ligands and amino acids from loop C. Such interactions were observed in both benzodiazepines^47^ and zolpidem.^48^ The carbonyl oxygen atoms of the tested compounds, including compound **6** (Figure 3), participate in hydrogen bonding interactions with the main chain of Ser-206 and the side chain of Ser-205. In addition, compound **6** exhibited hydrogen bond formation through its imidazo­[1,2-*a*]­pyridine nitrogen atom (interacting with Thr-207) and its 2,6-dimethylpiperazine component (interacting with Tyr-58). The amide group’s cyclic substituent structure subtly affected its interaction with Tyr-58, demonstrating a mild preference for molecules containing secondary amines.

To validate the stability and interaction mode of ligand **6** at the ECD of the GABAA receptor beyond static docking results, a 50 ns MD simulation was conducted. This approach enables the evaluation of ligand–receptor complex dynamics, specifically the duration of key interactions and conformational stability under physiological conditions. The small RMSD changes relative to the initial ligand pose (Figure 4) show that compound **6** fits well into the ECD and binds strongly to the surrounding amino acids. Despite its complex structure, the protein complex exhibits low variation in alpha-carbon positions. The 2-phenylimidazo­[1,2-*a*]­pyridine molecule binds stably within the hydrophobic pocket created by Phe-100, Tyr-210, Phe-77, and other residues. π-π stacking with Phe-77, evident in over 40% of our MD simulation frames, is one of the key factor stabilizing the complex in hydrophobic pocket. The imidazole ring’s nitrogen atom contribution, however, varies. The hydrogen bond with Thr-207 seen in the docking results is rapidly replaced by a water-mediated hydrogen bond to Tyr-160. Thirty percent of the simulation frames showed the molecule participating in the water hydrogen-bond network; however, this participation was sporadic in the latter stages. As anticipated, the hydrogen bond between Ser-205 in loop C and the ligand’s carbonyl group proved to be one of the most stable interactions. The simulation showed this occurring 95% of the time. Notably, the 2,6-dimethylpiperazine system exhibited interactions with Tyr-58 and Glu-189 amino acid residues. During the latter half of the simulation, stable cation-π and ionic interactions formed with Tyr-58 and Glu-189, respectively. This reinforces our earlier hypothesis that the observed activity differences stem primarily from how well the studied substituents match this amino acid. Glu-198’s involvement unveils a previously unrecognized interaction region for derivatives, presenting a promising direction for exploring zolpidem derivatives with basic substituents.

**Figure 4:**
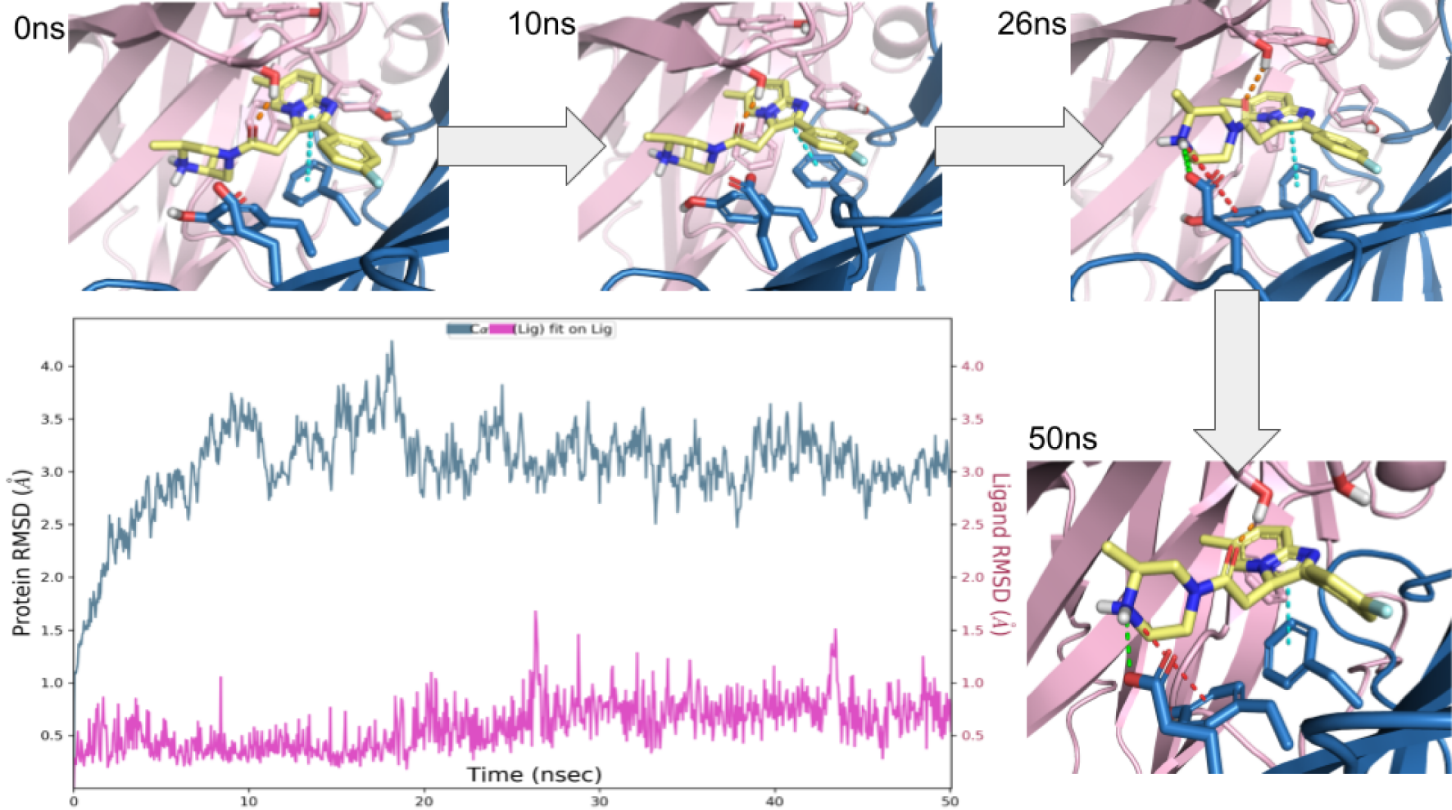
RMSD fluctuations, observed over a 50 ns MD simulation, reveals the dynamics of heavy atoms (pink plot) in molecule **6** and Cα atoms in GABAA (dark blue plot). Key simulation events are shown via visualizations of ligand-protein interactions. Dashed lines represent: hydrogen bonds (orange), cation-π interactions (red), aromatic CH-π and π-π interactions (cyan), and salt bridges (green).

Data from the experimental structure reveal that zolpidem binds not only to the α1/γ2 ECD, but also to the interface between the β2 and α1 transmembrane domains (TMDs). Despite the incomplete understanding of TMD sites’ role in zolpidem’s effects, we analyzed potential interactions of the studied compounds with this site.

As illustrated in Figure 5, molecule **6** is wholly contained within the hydrophobic environment of TMD. Aside from the salt bridge between the ionized secondary amine and Asp-282, no other specific interactions were observed. This binding mode was observed in all tested compounds, with only two exceptions. In compound **17**, a fluorophenyl group is oriented toward the receptor’s interior; hydrogen bonds, rather than ionic interactions, are formed with Asn-265 and the Ile-228 backbone. In contrast, compound **15** mimics zolpidem’s position by orienting its 1,3-thiazolidine system toward the exterior of the transmembrane domains.

**Figure 5:**
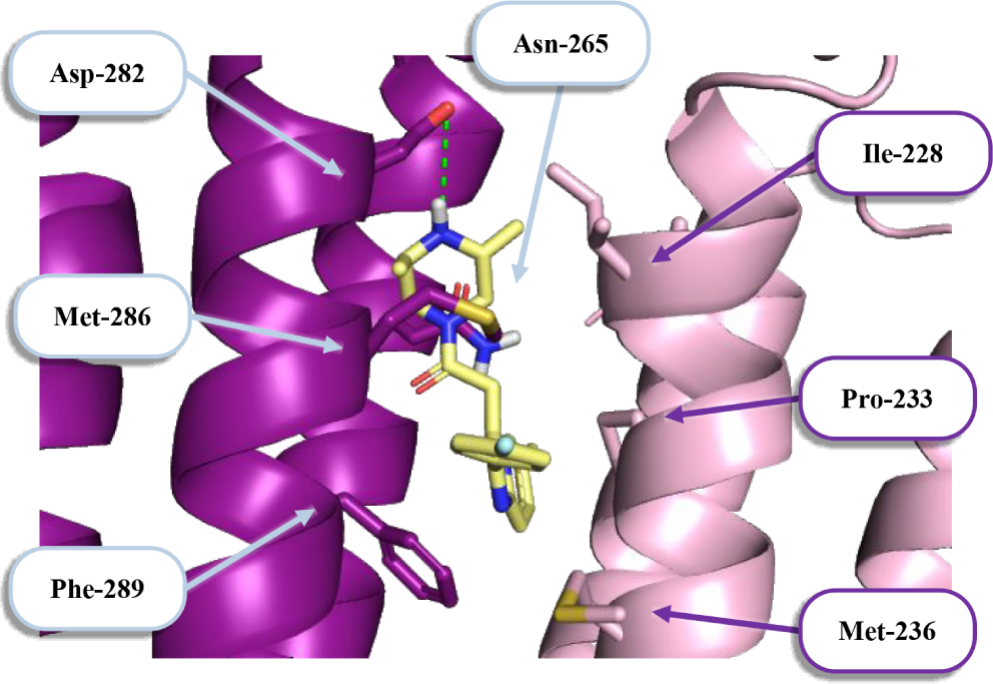
Binding mode of compound **6** (marked as yellow sticks) at the TMD interfaces of β2/α1 subunits. Chosen amino acids β2 subunit (highlighted in dark purple) and the α1-subunit (highlighted in pink) are marked with sticks in corresponding colors. Green dots indicate salt bridge.

### In Vitro Studies

#### Assessment of Neuroprotective Activities

Schizophrenia itself is associated with increased oxidative stress and neuronal damage. Patients with schizophrenia often exhibit structural brain abnormalities, such as reduced gray matter volume, suggesting a progressive loss of cerebral tissue.^49^ Other studies have consistently demonstrated that individuals with schizophrenia exhibit elevated markers of oxidative stress and decreased antioxidant defenses,^50–52^ implicating oxidative damage in the etiology and progression of the disorder.^53^ Therefore, neuroprotection has recently emerged as a novel trend in the development of potential therapeutic strategies for schizophrenia, aimed at preventing or mitigating the neuronal damage and loss that may contribute to the disorder’s progression and symptomatology.^54,55^ Building on previous reports highlighting the neuroprotective activities of GABA-A ligands,^38,39,56–59^ we evaluated the neuroprotective potential of our novel series.

Prior to determining the neuroprotective activity, we assessed the potential cytotoxicity of the selected set of compounds on the SH-SY5Y cell line and HepG2. No significant toxicity was observed at concentrations up to 100 μM (See Table S1 and S2). Next, the selected set of GABA-A ligands (compounds **6**, and **15**) was tested for their neuroprotective activities in a hydroxydopamine-induced neurotoxicity model using the human neuroblastoma SH-SY5Y cell line.^60^ As positive controls, we used zolpidem, known to protect neurons from various toxic insults,^38,56^ and the pyrrolidine derivative (**17**), which previously has demonstrated neuroprotective activity in similar *in vitro* models.^41^ All tested GABA-A ligands significantly improved cell survival following hydroxydopamine-induced injury (Figures 6 and 7), reduced intracellular ROS production (Figure 7B) and decreased the intracellular calcium levels, a hallmark of neuronal damage (Figure 7A). Compound **15**, a thiazolidine derivative, exhibited the most pronounced activity in reducing intracellular calcium levels, achieving up to a 50% reduction (Figure 7A). This effect was comparable to that of the reference drug zolpidem (47%) and the reference cyclic amine derivative, compound **17** (48%). The efficacy of 2,6-dimethylpiperazine (**6**), was slightly lower (28%), yet remained promising. Furthermore, we observed that the tested compounds also decreased the levels of ROS generated upon treatment of SH-SY5Y cells with hydrogen peroxide (Figure 7B). Under these conditions, compound **6**, a 2,6-dimethylpiperazine analogue, demonstrated the highest efficacy (32%).

**Figure 6:**
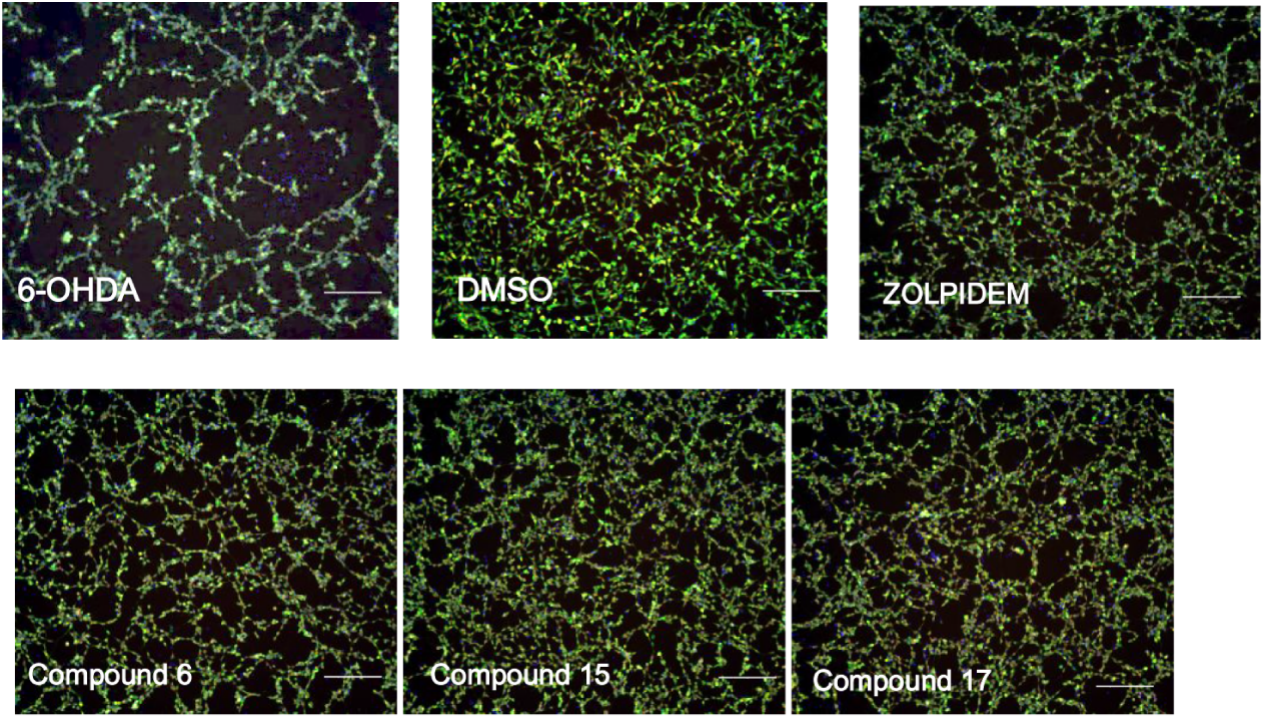
Representative images showing the neuroprotective effects of the tested compounds on human neuroblastoma SH-SY5Y cells. The cells were stained with Calcein AM to highlight the outer membranes and with Hoechst 33342 to detect the nucleus and Cell ROX to detect the intracellular ROS. 6-OHDA control: cells treated with 250 μM of 6-OHDA. addition (250 μM). DMSO control: cells treated with 0.1% DMSO only. Cells morphology assessment using a MultiWave Scoring module. The results of the experiment were captured using 10× magnification with the ImageXpress Micro XLS (Molecular Devices). Scale bar: 200 μm.

**Figure 7:**
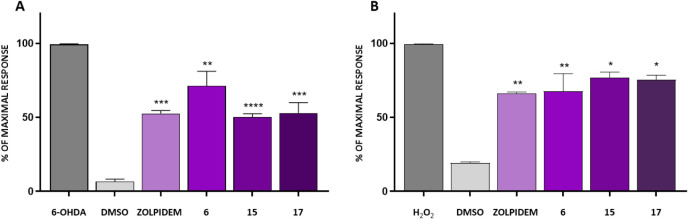
(A). Effect of 6-Hydroxydopamine (6-OHDA) on intracellular calcium concentration [Ca^2+^] determined by Fluo-4 AM. After pretreatment with tested compounds (50 μM) or vehicle (0.5% DMSO, v/v) for 1 h, the cells were incubated with 6-Hydroxydopamine (6-OHDA, 250 μM) for 1 h. 6-OHDA control: cells treated with 250 μM of 6-OHDA. addition (250 μM). DMSO control: cells treated with 0.5% DMSO only. Data are presented as the mean ± SD from 2–3 independent experiments. Experiments were carried on with ImageXpress Micro XLS (Molecular Devices). (B) The level of ROS generation was determined by Cell ROX dye. After pretreatment with tested compounds (50 μM) or vehicle (0.5% DMSO, v/v) for 1 h, the cells were incubated with hydrogen peroxide (H_2_O_2_, 300 μM) for 1 h. H_2_O_2_ control: cells treated with H_2_O_2_ (300 μM). DMSO control: cells treated with 0.5% DMSO only. Data are presented as the mean ± SD from 2–3 independent experiments. Experiments were carried on with the ImageXpress Micro XLS (Molecular Devices).

### In Vitro Metabolism and Extended Permeability Assay

At this stage, we selected a representative molecule from the series for further studies: the 2,6-dimethylpiperazine derivative (**6**), distinguished by its pronounced activity and high cell permeability (P_e_ = 16.8 ± 3.5 × 10^–6^ cm/s). Given that compounds bearing the 2-(4-fluorophenyl)-6-methylimidazo­[1,2-*a*]­pyridine scaffold^25,26^ have previously demonstrated optimal metabolic stability compared to their methyl counterparts,^35,36^ we began by confirming that our selected lead molecule **6**, maintains the required stability, unaffected by the presence of the 2,6-dimethylpiperazine moiety. Compound **6** was tested in the metabolic stability assay using human, mouse, and rat liver microsomes, as different species exhibit varying levels of metabolic enzymes and degradation specificity, which can lead to varying results. In general, compound **6** demonstrated high metabolic stability across liver microsomes from different species used in the assay. In human liver microsomes the t_0,5_ was >60 min, while in rodent liver microsomes the obtained t_0,5_ were t_0,5_ = 58 and 44 min for mouse and rat, respectively (Table 3 and Figures S1–S[Fig fig3]). The clearance values were also relatively low across all tested species, consistently remaining below 40 μL/mg/min, suggesting that the compound **6** is less likely to be rapidly metabolized or excreted. The main metabolic pathway was hydroxylation (M1), possibly within the 2-phenylimidazo­[1,2-*a*]-pyridine ring, as observed previously (Table 4).^25,26^ In rat liver microsomes, we also observed a small degree of dealkylated metabolite M2. Together, these data indicate that 2-phenylimidazo­[1,2-*a*]-pyridine derivatives possess suitable metabolic stability.

**Table 3:** Metabolic Stability of Compound **6** Assessed Using Different Microsomal Models: Human Liver Microsomes (HLMs), Mouse Liver Microsomes (MLMs), and Rat Liver Microsomes (RLMs)^a^

	% remaining of the parent compound			t_0,5_ [min]			Cl_int_ [μL/mg/min]		
Tested compound	HLMs	MLMs	RLMs	HLMs	MLMs	RLMs	HLMs	MLMs	RLMs
**6**	74	68	70	>60	58	44	<40	29.9	39.4

a Percentage of the parent compound remaining after 15 min of biotransformation, half-life (t_0,5_), and intrinsic clearance (Cl_int_) for Compound **6**.

**Table 4:** Biotransformation Pathways and Main Metabolites for Compound **6** Generated in the Presence of HLMs, MLMs, and RLMs

Metabolite	Percentage content among metabolites	Molecular mass of the metabolite (*m*/*z*)	Main metabolic pathway
HLMs			
M1	100%	397 [M+16]^+^	hydroxylation
MLMs			
M1	100%	397 [M+16]^+^	hydroxylation
RLMs			
M1	80%	397 [M+16]^+^	hydroxylation
M2	20%	244 [M-137]^+^	dealkylation

Although the preliminary pharmacological results for compound **6** were appealing, our subsequent studies revealed that **6** is a possible substrate for P-glycoprotein (P-gp). This was demonstrated using the MDCK-MDR1 permeability assay, a widely accepted model for studying P-gp-mediated efflux, in which cells overexpress the MDR1 gene encoding P-glycoprotein.^61^ We observed that the permeability values for compound **6** in the B-A assay were higher than those observed in the A-B permeability test (Table 4), reflecting a pattern similar to that of labetalol, a known reference drug and substrate for P-gp-mediated efflux.^62^ The calculated ratio of B-A/A-B permeability for compound **6** was 1.68, indicating a significant potential for P-gp efflux,^63,64^ which may further result in low BBB (blood-brain barrier) penetration. Given these considerations, compound **6** was not advanced to *in vivo* studies due to the risk of insufficient BBB penetration and the potential lack of *in vivo* efficacy resulting from it. At this stage, we selected compound **17**, a pyrrolidine derivative from our previous work,^41^ to investigate the potential antipsychotic properties of the cyclic amine series. In the MDCK-MDR1 permeability assay, compound **17** was determined not to be a substrate for P-gp efflux, as evidenced by its substantially lower B–A/A–B ratio = 0.58 (Table 5). Moreover, compound **17** possesses a proper ADMET, exhibits neuroprotective activities, and has already demonstrated efficacy in an animal model of ischemic stroke.^41^ Compound **17** has been also showed to be brain penetrant with *C*_max_: 219.5 [ng/g], AUC_0 → t_: 8229 [ng × min/g], t_0,5_= 269.6 min (see Table S3).^42^ This markedly prolonged half-life of 249 min represents a substantial improvement over zolpidem, whose elimination half-life has been reported as 30.6 min in rats and approximately 2 h in humans. Therefore, we reasoned that compound **17** would be a suitable candidate for an animal-level proof-of-concept study.

**Table 5:** A–B and B–A Permeability Assay in MDR1-MDCKII Cell Line (ph 7.4/7.4) for **6** and **17**

Permeability	Compound **6** (10 μM)	Compound **17** ^41^ (10 μM)	Labetalol
A–B [10^–6^ cm/s]	14.5	55.6	4.1
B–A [10^–6^ cm/s]	24.6	29.7	23.0
**Efflux ratio** ^a^	**1.68**	**0.53**	**5.6**

a The efflux ratio is expressed as P_aap_ B–A/A–B.

### In Vivo Studies

A set of behavioral tests was chosen for the pharmacological profiling of compound **17**, a representative of the novel series.^65,66^ In schizophrenia, dopaminergic striatal neurons are hyperreactive to amphetamine, leading to psychosis. Consequently, amphetamine-induced hyperlocomotion is a well-established rodent model for studying psychotic symptoms associated with dopaminergic hyperactivity.^67,68^ Compound **17** administrated *i.p.* significantly reduced amphetamine-induced hyperlocomotion at the doses of 3.0 mg/kg and 10.0 mg/kg (F­(3,28) = 15.0, *p* < 0.01, Figure 8A). Notably, the observed effect appeared to be specific, as no adverse changes in gross animal behavior were detected, nor was a reduction in spontaneous locomotor activity observed within the same dose range (see Figure 8C for comparison). These findings align with our previous studies, where we observed a significant reduction in drug-induced hyperlocomotion by α1β2γ2GABA-A receptor PAMs, including zolpidem^24^ and its structural derivatives.^25,26^ Our previous studies demonstrated that these effects are specific to the preferential PAM of α1β2γ2GABA-A receptors, in contrast to nonselective compounds like diazepam, which under similar conditions induce pronounced myorelaxant and ataxic effects, nearly abolishing spontaneous locomotor activity.^24^ The α1β2γ2GABA-A receptors are highly expressed in the basal ganglia, where they play a critical role in modulating dopaminergic transmission.^15^ A high binding density of the selective α1GABA-A PAM zolpidem has been identified in the rat globus pallidus,^28–30^ and studies suggest that selective α1GABA-A PAM modulators may regulate dopaminergic neurotransmission influencing motor functions and behavior.^33,34^ These findings suggest that the specific antipsychotic-like activity of compound **17** in the amphetamine-induced hyperlocomotion test may be associated with its modulatory action on α1β2γ2GABA-A receptors expressed in the basal ganglia.

**Figure 8:**
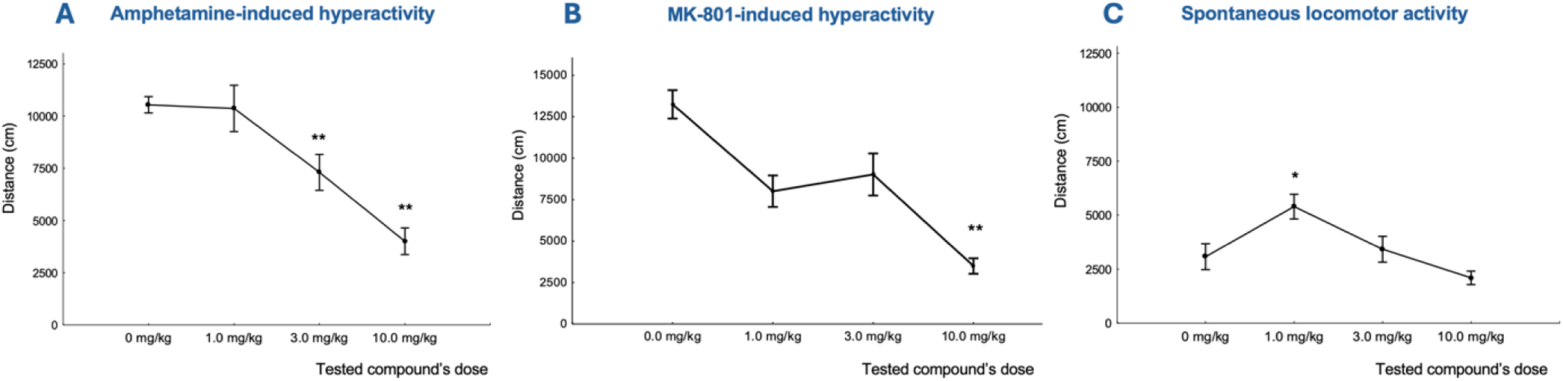
(A). Effects of compound **17**, administered at doses of 1–10 mg/kg (i.p.), on amphetamine-induced hyperlocomotion in rats. The dose of 0 mg/kg corresponds to subjects treated with vehicle and amphetamine (1.0 mg/kg, i.p.). Data are presented as the mean ± SEM (*n* = 8). One-way ANOVA F­(3,28)=0.2, *p* > 0.05, one-way ANOVA F­(3,28)=15.0, *p* < 0.01**. (B). Effects of compound **17** (1–10 mg/kg, i.p.) on MK-801-induced hyperlocomotion in rats. The dose of 0 mg/kg corresponds to subjects treated with vehicle and MK-801 (0.3 mg/kg, i.p.). Data are presented as the mean ± SEM (*n* = 8). ** *p* < 0.01, Newman-Keuls test, F­(3,28)=5.29, *p* = 0.005. (C) Effects of compound 9, administered at doses ranging from 1 to 10 mg/kg, on spontaneous locomotor activity in rats. Data are presented as the mean ± SEM (*n* = 8). One-way ANOVA F­(3,28) = 0.3, *p* > 0.05, one-way ANOVA F­(3,28) = 6.7, *p* < 0.01*.

Compound **17** was also evaluated in another test, the MK-801-induced hyperlocomotion model, a widely used tool for studying schizophrenia. MK-801, a noncompetitive NMDA receptor antagonist, is known to induce hyperlocomotion and mimic certain psychotic symptoms.^69^ MK-801 induces significant changes in neurochemistry and neuronal function, including disruption in dopamine-glutamate balance,^70^ increased glutamate release, which contributes to excitotoxicity and oxidative stress.^71^ In the MK-801-induced hyperlocomotion test, administration of compound **17** reduced hyperlocomotion across the dose range of 1 mg/kg, 3 mg/kg, and 10 mg/kg, with a significant effect observed at the 10 mg/kg dose (F­(3,28) = 5.29, *p* = 0.005, Figure 8B). The effect was specific, as compound **17** did not alter spontaneous locomotor activity within the same dose range (Figure 8C). We suspect that the observed antipsychotic effect of compound **17**, in the MK-801 test, may stem from its ability to enhance GABAergic signaling, thereby reducing the excitatory drive caused by increased glutamatergic transmission.

MK-801 was also employed in our studies to disrupt sensorimotor gating in rats, using the prepulse inhibition (PPI) test, a well-validated translational model for investigating sensorimotor gating in both humans and rodents.^72^ We observed that the tested compound **17** did not affect significantly basic startle response (F (3,28) = 1.66, *p* = 0.2, Figure 9A). Furthermore, compound **17** showed a tendency to attenuate MK-801-induced PPI deficits at a prestimulus of 84 and 90 dB, however, the effect did not reach statistical significance (F­(3,28) = 0.31, *p* = 0.82, Figure 9B and F (3,28) = 0.44, *p* = 0.72, Figure 9C). The lack of a significant effect of compound **17** on reversing MK-801-induced PPI deficits may be attributed to its specific GABA-A receptor subtype selectivity, as compound **17** is a highly specific α1 GABA-A positive allosteric modulator. According to literature data, the PPI responses are thought to be mediated by striatal and thalamic neuronal circuits, which are believed to be regulated via α3 GABA-A receptor subtypes.^73^ This is supported by evidence that α3 GABA-A receptor knockout (3KO) mice exhibit functional hyperactivity in the midbrain dopamine system and significant deficits in sensorimotor gating.^73^ Consequently, selective α3GABA-AR ligands have been suggested as a potential target in controlling the deficits in PPI in schizophrenia. Compound **17** demonstrates high efficacy at α1 GABA-A receptors (PAM efficacy: 201%) but limited efficacy at α3 GABA-A receptors (PAM efficacy: 139%, baseline GABA response: 110%). In our view, this receptor selectivity likely precludes its ability to exhibit *in vivo* efficacy in the PPI model, where α3 GABA-A PAMs are typically more effective.^41^

**Figure 9:**
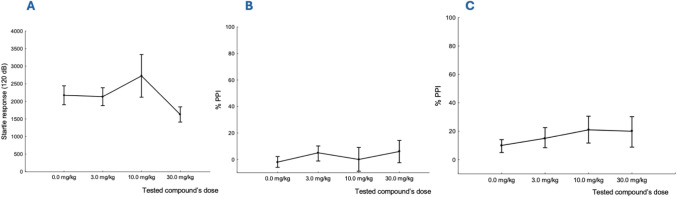
(A). The effects of tested compound **17** on basic startle response. (B). The effects of the tested compound on MK801-induced deficit in PPI evoked by a prestimulus of 84 dB F­(3,28) = 0.31, *p* = 0.82, and (C). by prestimulus of 90 dB (F­(3,28) = 0.44, *p* = 0.72).

## Conclusions

Clinical studies have identified significant reductions in GABAergic markers within the nigrostriatal pathways of patients with schizophrenia, potentially contributing to dysregulated dopamine signaling and the onset of psychotic symptoms.^15,17^ Previous experimental studies have demonstrated that α1β2γ2 GABA-A receptor PAMs are effective in mitigating psychotic symptoms, highlighting this mechanism as an attractive molecular target for the development of antipsychotic agents with a nondopaminergic mode of action.^24–26^ Given the limited availability of α1β2γ2 GABA-A ligands, we explored the chemical space of 2-phenylimidazo­[1,2-*a*]-pyridine derivatives bearing cyclic amine amides to identify potent α1β2γ2 GABA-A receptor PAMs with improved pharmacokinetic profile as potential treatments for psychotic symptoms in schizophrenia. Our studies found that incorporating cyclic amine moieties into the amide end of the 2-phenylimidazo­[1,2-*a*]-pyridine core yields highly active compounds at α1β2γ2GABA-A receptor. The compounds were evaluated through a series of pharmacological assays, from which we identified the most promising molecule, a 2,6-dimethylpiperazine analogue (**6**). This compound demonstrated potent PAM efficacy at the α1β2γ2 GABA-A receptor, neuroprotective activity, and exhibited relatively high metabolic stability *in vitro*. However, despite promising preclinical data, further studies revealed that compound **6** was prone to P-gp efflux, thereby posing a risk of low BBB penetration. These findings led us to investigate another representative of the cyclic amine series, compound **17**, which exhibits high PAM efficacy at α1β2γ2 GABA-A receptors, demonstrates the ability to suppress hydroxylamine-induced toxicity, is not a substrate for P-gp efflux and possess improved farmakokinetic profile comparing to zolpidem. *In vivo* studies revealed that compound **17** exhibits a distinctive pharmacological profile with antipsychotic efficacy, as evidenced by its ability to attenuate amphetamine- and MK-801-induced hyperlocomotion. Notably, these effects were selective and did not alter spontaneous locomotor activity at the same doses. We propose that the observed antipsychotic activity may plausibly be attributed to the unique ability of compound **17** to modulate α1β2γ2 GABA-A receptors, which are highly expressed in the basal ganglia and are involved in the regulation of dopaminergic signaling.^28,30^ At the same time, compound **17** did not restore disrupted sensorimotor gating, which we attribute to its lack of interaction with α3GABA-A receptor subtypes. This distinct pharmacological profile arises from the specific subunit selectivity of GABA-A receptors, as compound **17** is a selective α1GABA-A PAM with minimal interaction with the α3GABA-A receptor subtype. Our findings highlight α1β2γ2 GABA-A PAMs as a promising therapeutic strategy for schizophrenia-related psychosis, offering a nondopaminergic mechanism of action. Notably, these ligands also exhibit neuroprotective activity, adding an additional layer of therapeutic potential. These two properties distinguish α1β2γ2 GABA-A ligands as promising candidates for the treatment of schizophrenia.

## Methods

### Molecular Modeling

Molecular modeling simulations were performed with Maestro Schrödinger (Release 2021-2). Ligand structures were prepared and optimized using the LigPrep tool with OPLS4 force field. Epik was used for charges calculation at pH 7.4. All possible stereoisomers and tautomers were generated for each ligand.

From the PDB database (PDB ID code: 8DD2), the human GABA-A receptor α1β2γ2 subtype complex in complex with GABA and Zolpidem was retrieved.^48^ Following the addition of hydrogen atoms, the complex’s disulfide bonds were restored, and its hydrogen bond network was optimized via PROPKA at a pH of 7.4. Initial energy minimization employed the OPLS4 algorithm with heavy atom converge RMSD 0.5 Å. Refinement of the binding sites was achieved using the Refine Protein–Ligand Complex tool, employing a local optimization method that considered flexible protein residues within a 7 Å radius of the Zolpidem molecules and the VSGB solvation model. A separate molecular docking grids were generated, centered on the Zolpidem molecule at extracellular domain and at α1/β2 transmembrane domain. The grid size was fitted to include ligands of comparable proportions, and to allow for rotation around all hydroxyl and sulfhydryl groups. The docking process employed the SP protocol, incorporating preoptimized ring conformations of ligands. Both intramolecular and aromatic hydrogen bonds were set to influence the final docking score.

Molecular dynamics (MD) simulations were performed using Desmond^74^ for complex of GABAA with 6 compound obtained after docking. Sodium ions were used to neutralize systems within orthorhombic simulation boxes, which were then solvated using the TIP4P solvent model and 150 mM NaCl. The complex was incorporated within the POPE membrane. The ensemble class NPγT was utilized during the MD simulation. Temperature was set at 310.2 K, and the pressure was 1 bar. The visualizations were prepared in PyMol (PyMOL Molecular Graphics System, Version 2.4.1, Schrödinger, LLC).

### General Chemistry Information

Unless stated otherwise, all chemicals were obtained from commercial sources and used without further purification. Microwave-assisted synthesis was conducted by CEM-Discover system operating at 2.445 GHz. The progress of reactions and purity of products were evaluated and monitored by TLC (aluminum plates coated with silica gel 60 F254, purchased from Merck), visualized using 254 nm UV lamp. Obtained compounds were purified (over 95% purity by HPLC for all final products) via chromatographic methods. Manual column chromatography (silica gel RediSep, particle size 40–60 μm 60 Å) separations were performed with DCM/MeOH system as an eluent, ranging MeOH concentration from 2% to 10% [*v/v*]. Automated flash chromatography was performed using CombiFlash RF (Teledyne Isco) system, equipped with either RediSep Gold columns (silica gel 60, particle size 20–40 μm), or RediSep R_f_ flash columns (silica gel 60, particle size 40–63 μm). Preparative HPLC purifications employed a Jasco LC-400 system with a Phenomenex Luna C8 column (5 μm, 15 mm × 21.2 mm). All ^1^H NMR, ^13^C NMR and ^19^F NMR data was collected with either JEOL JNM-ECZR 500 RS1 (ECZR version) at frequencies of 500, 126, and 471 MHz, or Varian Mercury spectrometer (Varian Inc., Palo Alto, CA, USA) at frequencies of 300 MHz, 75 and 282 MHz. Chemical shifts (δ) in all NMR spectra were calibrated against the reference deuterated solvents (CDCl_3_, CD_3_OD, (CD_3_)_2_SO) and presented as parts per million (ppm). The UPLC–MS spectrometry was performed using Waters ACQUITY UPLC (Waters Corporation, Milford, MA, USA) combined with a Waters tandem quadrupole mass spectrometer (TQD) via electrospray ionization (ESI) mode, with respect to the protocols published before.^42^ All of the spectral data was analyzed with the ACD/Spectrus Processor 2017 software.

#### 2-(4-Fluorophenyl)-6-methylimidazo­[1,2-*a*]­pyridine (**1**)

5-methylpyridine-2-amine (1 eq, 9 mmol), 2-bromo-1-(4-fluorophenyl)­ethan-1-one (1 eq, 9 mmol) and sodium bicarbonate (1.5 eq, 13.5 mmol) in toluene solution (15 mL) were stirred for 1 h at 100 °C under continuous microwave irradiation (200 W). Afterward, resulting precipitate was filtered off, washed with petroleum ether, distilled water and diethyl ether. Drying under reduced pressure gave a beige solid with 91% yield and no need for additional purification. ^1^H NMR (CDCl_3_, 500 MHz) δ 7.88 (dd, 3H, *J* = 5.4, 9.0 Hz), 7.69 (s, 1H), 7.50 (d, 1H, *J* = 9.2 Hz), 7.09 (t, 2H, *J* = 8.8 Hz), 7.00 (dd, 1H, *J* = 1.6, 9.2 Hz), 2.30 (s, 3H). ^13^C NMR (CDCl_3_, 126 MHz) δ 163.4 (d, *J* = 248.7 Hz), 146.59, 144.9, 130.3 (2C), 128.0, 127.7, 123.4, 122.2, 116.9 (d, *J* = 20.9 Hz) (2C), 115.8, 107.6, 21.4. ^19^F NMR (CDCl_3_, 471 MHz) δ – 114.4 (s, 1F). Formula: C_14_H_11_FN_2_; MS (ESI^+^) *m*/*z* 227.024 (M+H^+^), calculated: 227.098 (protonated).

#### 2-(2-(4-Fluorophenyl)-6-methylimidazo­[1,2-*a*]­pyridin-3-yl)-2-hydroxyacetic Acid (**2**)

2-(4-fluorophenyl)-6-methylimidazo­[1,2-*a*]­pyridine (**1**) (1 eq, 8.2 mmol) and glyoxylic acid (2.6 eq, 21.3 mmol) were dissolved in DCM (25 mL) and stirred under reflux for 16 h. The solvent was then evaporated, and the crude product was recrystallized from methanol, filtered and dried to result in a white powder. Compound **2** was obtained with 90% yield.^1^ H NMR (CDCl_3_, 500 MHz) δ 8.29–8.3 (s, 1H), 7.82–7.79 (s, 2H), 7.53–7.51 (d, 1H), 7.30 (t, 2H), 7.16–7.18 (m, 1H), 5.63 (s, 1H), 2.3 (s, 3H). ^13^C NMR (CDCl_3_, 126 MHz) δ 163.47, 161.52 (d, *J* = 248.1 Hz), 143.69, 142.76, 131.09 (2C), 128.85, 124.24, 121.90, 118.56, 116.77, 116.08, 115.91 (d, *J* = 21.1 Hz) (2C), 64.78, 18.45. ^19^F NMR (CDCl_3_, 471 MHz) δ −114.12 (s, 1F). Formula: C_16_H_13_FN_2_O_3_; MS (ESI^+^) *m*/*z* 301.214 (M+H^+^), calculated: 301.099 (protonated).

#### 2-(2-(4-Fluorophenyl)-6-methylimidazo­[1,2-*a*]­pyridin-3-yl)­acetic Acid (**3**)

Compound (**2**) (1 eq, 6 mmol) was stirred with Pd/C (0.1 eq, 0.6 mmol) in formic acid (20 mL) at 100 °C for 16 h. The mixture was then cooled down and subsequently diluted with methanol. Filtration through silica gel and solvent evaporation gave a yellowish solid with 93% yield. Additional purification was unnecessary. ^1^H NMR ((CD_3_)_2_SO, 500 MHz) δ 8.23 (s, 1H), 8.16 (s, 1H), 7.81–7.78 (s, 2H), 7.46–7.44 (d, 1H), 7.25 (t, 2H), 7.10–7.08 (d, 1H), 3.93 (s, 2H), 2.26 (s, 3H). ^13^C NMR ((CD_3_)_2_SO, 126 MHz) δ 171.90, 161.14 (d, *J* = 248.08 Hz), 143.29, 141.59, 131.74, 130.06 (2C), 127.83, 122.90 (d, *J* = 3.6 Hz), 121.47, 116.49, 115.99 (d, *J* = 21.1 Hz) (2C), 115.82 (d, *J* = 19.31 Hz), 31.63, 18.33. ^19^F NMR ((CD_3_)_2_SO, 471 MHz) δ-114.99 (s, 1F). Formula: C_16_H_13_FN_2_O_2_; MS (ESI^+^) *m*/*z* 285.210 (M+H^+^), calculated: 285.104 (protonated).

### General Procedure for Preparation of Compounds (**4–17**)

Appropriate volume (3 mL for 100 mg of core acid **3**) of freshly distilled anhydrous tetrahydrofuran was used to suspend the core acid (**3**) (1 eq, 0.7 mmol) under argon. The mixture was treated with ultrasounds for about 10 min and then cooled to 10 °C in ice bath for slow addition of *N,N*’-carbonyldiimidazole (CDI) (1.3 eq, 0.91 mmol). The solution was then stirred to activate the acid at room temperature for about 1 h, with HPLC monitoring. After successful activation of the acid, a selected amine (1.2 eq, 0.84 mmol) was added. Reaction mixture was then heated to 40 °C and stirred for 12 h in inert atmosphere. The solvent was then evaporated and the final product was isolated via column chromatography, as specified in general chemistry information section.

#### 2-[2-(4-Fluorophenyl)-6-methylimidazo­[1,2-*a*]­pyridin-3-yl]-1-(4-methylpiperazin-1-yl)­ethan-1-one formate salt (**4**)

The core acid (**3**) was coupled with 1-methylpiperazine according to the general procedure, yield: 58%. Pure formate salt was formed due to the need of additional purification via preparative HPLC (10–60% acetonitrile in water with 0.05% formic acid). ^1^H NMR (CDCl_3_, 500 MHz) δ 8.24 (s, 1H), 8.09 (s, 1H), 7.64 (d, 1H, *J* = 9.2 Hz), 7.57–7.61 (m, 2H), 7.21 (dd, 1H, *J* = 1.6, 9.2 Hz), 7.16 (t, 2H, *J* = 8.7 Hz), 6.41 (br s, 1H), 4.12 (s, 2H), 3.81 (m, 2H), 3.57 (br t, 2H, *J* = 4.8 Hz), 2.70 (m, 2H), 2.55 (m, 2H), 2.47 (s, 3H), 2.39 (s, 3H). ^13^C NMR (CDCl_3_, 126 MHz, single pulse quintuple) δ 166.3, 164.8, 162.8, 143.4, 141.2, 130.3 (2C), 129.3, 129.0, 123.3, 122.4, 116.0 (2C), 115.9, 113.7, 54.0, 53.9, 44.8, 44.5, 40.8, 29.7, 18.5.^19^ F NMR (CDCl_3_, 471 MHz) δ −111.96 (s, 1F). Formula: C_21_H_23_FN_4_O•HCOOH; MS (ESI^+^) *m*/*z*: 376.363 (M + H^+^), calculated: 367.193 (protonated).

#### 2-[2-(4-Fluorophenyl)-6-methylimidazo­[1,2-*a*]­pyridin-3-yl]-1-(piperazin-1-yl)­ethan-1-one (**5**)

The core acid (**3**) was coupled with piperazine according to the general procedure. Compound (**5**) was obtained as a white solid, yield: 26%. Spectral data matched those reported previously:^41^ ^1^H NMR (CDCl_3_, 300 MHz) δ 7.9–8.1 (m, 2H), 7.6–7.7 (m, 2H), 7.52 (dd, 1H, *J* = 0.9, 9.1 Hz), 7.19 (br t, 2H, *J* = 8.6 Hz), 7.1–7.1 (m, 1H), 4.13 (s, 2H), 3.61 (br dd, 1H, *J* = 4.5, 6.0 Hz), 3.5–3.6 (m, 1H), 3.3–3.5 (m, 2H), 3.1–3.3 (m, 4H), 2.9–3.0 (m, 1H), 2.37 (s, 3H). ^13^C NMR (CDCl_3_, 75 MHz) δ ppm 166.77, 162.60 (d, *J* = 248.2 Hz), 160.76, 144.33 (d, *J* = 1.7 Hz), 142.85, 130.10 (d, *J* = 8.3 Hz) (2C), 128.03, 122.2 (d, *J* = 6.1 Hz), 116.8, 116.70 (d, *J* = 21.6 Hz) (2C), 112.83, 45.35, 42.59, 41.5, 39.86, 30.22, 18.46. ^19^F NMR (CDCl_3_, 282 MHz) δ −113.30 (s, 1 F). Formula: C_20_H_21_FN_4_O; MS (ESI^+^) *m*/*z* 353.125 (M+H^+^), calculated: 353.178 (protonated).

#### 1-(3,5-Dimethylpiperazin-1-yl)-2-[2-(4-fluorophenyl)-6-methylimidazo­[1,2-*a*]­pyridin-3-yl]­ethan-1-one (**6**)

The core acid (**3**) was coupled with 2,6-dimethylpiperazine according to the general procedure. Compound (**6**) was obtained as a white solid, yield: 62%. ^1^H NMR (CDCl_3_, 500 MHz) δ 8.10 (s, 1H), 7.65 (dd, 2H, *J* = 5.4, 8.7 Hz), 7.53 (d, 1H, *J* = 9.0 Hz), 7.18 (t, 2H, *J* = 8.7 Hz), 7.09 (dd, 1H, *J* = 1.6, 9.2 Hz), 5.30 (s, 1H), 4.47 (br d, 1H, *J* = 12.7 Hz), 4.04–4.19 (m, 2H), 3.40–3.46 (m, 1H), 2.67–2.76 (m, 1H), 2.53–2.66 (m, 1H), 2.44–2.52 (m, 1H), 2.36 (s, 3H), 2.25–2.34 (m, 1H), 1.13 (br d, 3H, *J* = 5.9 Hz), 0.87 (br d, 3H, *J* = 6.2 Hz). ^13^C NMR (CDCl_3_, 126 MHz, single pulse quintuple) δ 166.3, 162.6, 144.3, 142.4, 130.7, 130.2 (2C), 128.0, 122.3, 122.2, 116.7, 115.8 (2C), 113.1, 53.4, 51.4, 51.1 (2C), 30.4 (2C), 18.5 (2C). ^19^F NMR (CDCl_3_, 471 MHz) δ −113.62 (s, 1F). Formula: C_22_H_25_FN_4_O; MS (ESI^+^) *m*/*z* 381.220 (M+H^+^), calculated: 381.209 (protonated).

#### 2-[2-(4-Fluorophenyl)-6-methylimidazo­[1,2-*a*]­pyridin-3-yl]-1-(4-methylpiperidin-1-yl)­ethan-1-one (**7**)

The core acid (**3**) was coupled with 4-methylpiperidine according to the general procedure. Compound (**7**) was obtained as a white solid, yield: 14%. 1H NMR (CDCl3, 500 MHz) δ 8.03 (s, 1H), 7.63 (dd, 2H, J = 5.4, 8.7 Hz), 7.54 (d, 1H, J = 9.2 Hz), 7.16 (t, 2H, J = 8.7 Hz), 7.07 (dd, 1H, J = 1.6, 9.2 Hz), 4.52 (td, 1H, J = 1.9, 13.2 Hz), 4.00–4.14 (m, 2H), 3.61–3.64 (m, 1H), 2.86 (dt, 1H, J = 2.7, 13.0 Hz), 2.56 (dt, 1H, J = 2.9, 12.8 Hz), 2.36 (s, 3H), 1.64 (br dd, 1H, *J* = 2.0, 13.4 Hz), 1.51–1.57 (m, 1H), 1.46–1.49 (m, 1H), 0.94–1.03 (m, 1H), 0.89 (d, 3H, *J* = 6.5 Hz), 0.70–0.78 (m, 1H). ^13^C NMR (CDCl_3_, 126 MHz) δ 166.3, 162.5 (d, *J* = 247.3 Hz), 144.1, 142.6, 130.8 (d, *J* = 3.3 Hz), 130.2 (d, *J* = 8.1 Hz) (2C), 127.8, 122.2, 122.0, 116.6, 115.6 (d, *J* = 21.4 Hz) (2C), 114.0, 46.3, 42.6, 34.5, 33.8, 30.8, 30.2, 21.6, 18.5. ^19^F NMR (CDCl_3_, 471 MHz) δ −114.08 (s, 1F). Formula: C_22_H_24_FN_3_O; MS (ESI^+^) *m*/*z* 366.147 (M+H^+^), calculated: 366.460 (protonated base).

#### 2-[2-(4-Fluorophenyl)-6-methylimidazo­[1,2-*a*]­pyridin-3-yl]-1-[4-(propan-2-yl)­piperazin-1-yl]­ethan-1-one (**8**)

The core acid (**3**) was coupled with 1-(propan-2-yl)­piperazine according to the general procedure. Compound (**8**) was obtained as a white solid, yield: 7%. 1H NMR (CDCl3, 500 MHz) δ 8.01 (s, 1H), 7.63 (dd, 2H, J = 5.4, 8.7 Hz), 7.54 (d, 1H, J = 9.2 Hz), 7.16 (t, 2H, J = 8.7 Hz), 7.08 (dd, 1H, J = 1.6, 9.2 Hz), 4.07 (s, 2H), 3.62 (br s, 2H), 3.34 (br t, 2H, *J* = 4.9 Hz), 2.66–2.69 (m, 1H), 2.44 (br t, 2H, *J* = 4.8 Hz), 2.37 (s, 3H), 2.23–2.25 (m, 2H), 1.00 (d, 6H, *J* = 6.6 Hz). ^13^C NMR (CDCl3, 126 MHz) δ 166.5, 162.6 (d, J = 247.4 Hz), 144.2, 142.8, 130.8 (d, J = 3.3 Hz), 130.2 (d, J = 8.2 Hz) (2C), 127.8, 122.1, 122.0, 116.7, 115.7 (d, J = 21.4 Hz) (2C), 113.7, 66.8, 54.5 (2C), 48.6 (2C), 48.2, 29.9, 18.5, 18.2. ^19^F NMR (CDCl_3_, 471 MHz) δ −113.34 (s, 1F). Formula: C_23_H_27_FN_4_O; MS (ESI^+^) *m*/*z* 395.306 (M+H^+^), calculated: 395.502 (protonated base).

#### 1-(4-Cyclopropylpiperazin-1-yl)-2-[2-(4-fluorophenyl)-6-methylimidazo­[1,2-*a*]­pyridin-3-yl]­ethan-1-one (**9**)

The core acid (**3**) was coupled with 1-cyclopropylpiperazine according to the general procedure. Compound (9) was obtained as a white powder, yield: 20%. ^1^H NMR (CDCl3, 500 MHz) δ 8.08 (br s, 1H), 7.63–7.67 (m, 3H), 7.16–7.20 (m, 3H), 4.10 (s, 2H), 3.58–3.69 (m, 2H), 3.32–3.44 (m, 2H), 2.57–2.67 (m, 2H), 2.44–2.55 (m, 2H), 2.39 (s, 3H), 0.83–0.84 (m, 1H), 0.47–0.55 (m, 4H). ^13^C NMR (CDCl_3_, 126 MHz, single pulse quintuple) δ ppm 166.3, 162.7, 143.4, 141.4, 130.3, 129.5, 128.8, 122.8, 122.4, 116.1, 115.8, 114.0, 53.2, 52.9, 45.7, 41.9, 38.3, 29.8, 18.4, 5.8. ^19^F NMR (CDCl3, 471 MHz) δ −112.53 (s, 1F). Formula: C_23_H_25_FN_4_O; MS (ESI^+^) *m*/*z* 393.370 (M+H^+^), calculated: 393.486 (protonated base).

#### 2-[2-(4-Fluorophenyl)-6-methylimidazo­[1,2-*a*]­pyridin-3-yl]-1-[4-(2-methylpropyl)­piperazin-1-yl]­ethan-1-one (**10**)

The core acid (**3**) was coupled with 1-(2-methylpropyl)­piperazine according to the general procedure. Compound (**10**) was obtained as a white solid, yield: 22%. ^1^H NMR (CDCl_3_, 500 MHz) δ 7.98 (s, 1H), 7.61 (dd, 2H, *J* = 5.4, 8.9 Hz), 7.52 (d, 1H, *J* = 9.0 Hz), 7.14 (t, 2H, *J* = 8.7 Hz), 7.06 (dd, 1H, *J* = 1.6, 9.2 Hz), 4.03 (s, 2H), 3.6–3.6 (m, 2H), 3.3–3.3 (m, 2H), 2.34 (s, 3H), 2.31 (br t, 2H, *J* = 5.0 Hz), 2.14 (br t, 2H, *J* = 5.0 Hz), 2.03 (d, 2H, *J* = 7.3 Hz), 1.72 (m, 1H), 0.86 (d, 6H, *J* = 6.6 Hz). ^13^C NMR­(CDCl_3_, 126 MHz, single pulse quintuple) δ 166.4, 162.5, 144.0, 142.5, 130.5, 130.1 (2C), 127.9, 122.1 (2C), 116.5, 115.6 (2C), 113.7, 66.4, 53.5, 53.0, 45.8, 42.0, 29.8, 25.2, 20.7 (2C), 18.4. ^19^F NMR (CDCl_3_, 471 MHz) δ −113.85 (s, 1F). Formula: C_24_H_29_FN_4_O; MS (ESI^+^) *m*/*z* 409.133 (M+H^+^), calculated: 409.529 (protonated base).

#### 1-(4-*tert*-Butylpiperazin-1-yl)-2-[2-(4-fluorophenyl)-6-methylimidazo­[1,2-*a*]­pyridin-3-yl]­ethan-1-one (**11**)

The core acid (**3**) was coupled with 1-*tert*-butylpiperazine according to the general procedure. Compound (**11**) was obtained as a white powder, yield: 13%. ^1^H NMR (CDCl3, 500 MHz) δ = 8.01 (s, 1H), 7.65–7.62 (m, 2H), 7.53 (d, *J* = 9.2 Hz, 1H), 7.18 (br t, *J* = 8.5 Hz, 2H), 7.09 (br d, *J* = 9.2 Hz, 1H), 4.09–4.04 (m, 2H), 3.72–3.64 (m, 2H), 3.50–3.46 (m, 2H), 2.59–2.48 (m, 2H), 2.35 (s, 3H), 2.23–2.12 (m, 2H), 1.25 (s, 9H). ^13^C NMR (CDCl_3_, 126 MHz, single pulse quintuple) δ = 166.4, 162.6, 144.2, 142.6, 130.6, 130.5, 130.2, 128.1, 122.4, 122.1, 116.7, 115.9, 45.7, 29.8, 25.3, 25.1, 24.8, 18.4. ^19^F NMR (CDCl3, 471 MHz) δ = −113.86 – −113.99 (m, 1F). Formula: C_24_H_29_FN_4_O; MS (ESI^+^) *m*/*z* 409.333 (M+H^+^), calculated: 409.529 (protonated base).

#### 2-[2-(4-Fluorophenyl)-6-methylimidazo­[1,2-*a*]­pyridin-3-yl]-1-(octahydro-1H-isoindol-2-yl)­ethan-1-one (**12**)

The core acid **(3**) was coupled with octahydro-1H-isoindole according to the general procedure. Compound (**12**) was obtained as a white solid, yield: 6%. 1H NMR (CD3OD, 500 MHz) δ 8.07 (s, 1H), 7.63 (dd, 2H, J = 5.4, 8.8 Hz), 7.48 (dd, 1H, J = 0.7, 9.2 Hz), 7.20–7.25 (m, 3H), 4.07–4.16 (m, 2H), 3.58 (dd, 1H, J = 7.2, 10.3 Hz), 3.42–3.47 (m, 2H), 35.3–3.39 (m, 1H), 2.24–2.28 (m, 4H) (signals overlap), 2.24–2.28 (m, 1H), 1.41–1.69 (m, 8H). ^13^C NMR (CD3OD, 126 MHz) δ 170.1, 164.3 (d, J = 246.2 Hz), 145.3, 143.7, 131.9 (d, J = 3.3 Hz), 131.6 (d, J = 8.2 Hz) (2C), 130.0, 124.0, 123.8, 116.80, 116.63 (2C), 116.44, 51.9, 51.6, 39.1, 37.5, 31.1, 27.0, 26.7, 24.0, 23.7, 18.4. ^19^F NMR (CD_3_OD, 471 MHz) δ −115.97 (s, 1F). Formula: C_24_H_26_FN_3_O; MS (ESI^+^) *m*/*z* 392.088 (M+H^+^), calculated: 392.498 (protonated base).

#### 1-(Azepan-1-yl)-2-(2-(4-fluorophenyl)-6-methylimidazo­[1,2-*a*]­pyridin-3-yl)­ethan-1-one (**13**)

The core acid (**3**) was coupled with azepane according to the general procedure. Compound (**13**) was obtained as a white powder. ^1^H NMR (CDCl_3_, 500 MHz) δ = 8.05 (s, 1H), 7.64 (dd, J = 5.4, 8.7 Hz, 2H), 7.53 (d, J = 9.2 Hz, 1H), 7.16 (t, J = 8.7 Hz, 2H), 7.07 (dd, J = 1.7, 9.2 Hz, 1H), 4.09 (s, 2H), 3.53–3.51 (m, 2H), 3.30 (t, J = 5.7 Hz, 2H), 2.36 (d, J = 0.7 Hz, 3H), 1.71–1.67 (m, 2H), 1.51–1.46 (m, 6H). ^13^C NMR (CDCl_3_, 126 MHz) δ 167.7, 162.5, 144.2, 142.8, 130.9, 130.2, 127.7, 122.3, 121.9, 116.6, 115.6, 114.0, 48.1, 46.6, 30.5, 29.0, 27.3, 27.0, 26.5, 18.5. ^19^F NMR (CDCl3, 471 MHz) δ −114.19 (s, 1F). Formula: C_22_H_24_FN_3_O; MS (ESI^+^) *m*/*z* 366.280 (M+H^+^), calculated: 366.460 (protonated base).

#### 1-(2,2-Dimethyl-1,3-thiazolidin-3-yl)-2-[2-(4-fluorophenyl)-6-methylimidazo­[1,2-*a*]­pyridin-3-yl]­ethan-1-one (**14**)

The core acid (**3**) was coupled with 2,2-dimethyl-1,3-thiazolidine according to the general procedure. Compound (**1**4) was obtained as a white powder, yield: 18%. ^1^H NMR (CDCl3, 500 MHz) δ 7.99 (br s, 1H), 7.84–7.87 (m, 1H), 7.67 (br dd, 2H, *J* = 5.2, 8.4 Hz), 7.31–7.36 (m, 1H), 7.18 (br t, 2H, *J* = 8.2 Hz), 4.10 (s, 2H), 3.89–3.91 (m, 2H), 3.01–3.03 (m, 2H), 2.41 (s, 3H), 1.83 (s, 6H). ^13^C NMR (CDCl_3_, 126 MHz, single pulse quintuple) δ 165.1, 162.8, 142.5, 140.4, 130.3, 129.8, 128.3, 123.6, 122.2, 115.9, 115.6, 114.3, 72.9, 53.1, 33.3, 28.4, 27.9, 18.4. ^19^F NMR (CDCl3, 471 MHz) δ −111.16 (s, 1F). Formula: C_21_H_22_FN_3_OS; MS (ESI^+^) *m*/*z* 384.191 (M+H^+^), calculated: 384.493 (protonated base).

#### 2-[2-(4-Fluorophenyl)-6-methylimidazo­[1,2-*a*]­pyridin-3-yl]-1-(1,3-thiazolidin-3-yl)­ethan-1-one (**15**)

The core acid (**3**) was coupled with 1-methylpiperazine according to the general procedure. Compound (**15**) was obtained as a white solid, yield: 65%. ^1^H NMR (CD_3_OD, 500 MHz) δ 8.04 (s, 1 H), 7.61 (m, 2 H), 7.46–7.49 (m, 1 H), 7.18–7.25 (m, 3 H), 4.68 (s, 1 H), 4.57 (s, 1 H), 4.21 (s, 1 H), 4.18 (s, 1 H), 3.89 (t, 1 H, *J* = 6.2 Hz), 3.81 (t, 1 H, *J* = 6.4 Hz), 3.17 (t, 1 H, *J* = 6.2 Hz), 3.07 (t, 1 H, *J* = 6.4 Hz), 2.36 (s, 3 H). ^13^C NMR (CD_3_OD, 126 MHz, single pulse quintuple) δ 169.1, 168.9, 164.3, 145.3, 143.8, 131.7, 131.6, 130.1, 124.2, 123.7, 116.8, 116.6, 116.1 (2C), 32.1, 31.8, 31.4, 30.3, 18.3. ^19^F NMR (CD_3_OD, 471 MHz) δ −115.93 (s, 1F). Formula: C_19_H_18_FN_3_OS; MS (ESI^+^) *m*/*z* 356.056 (M+H^+^), calculated: 356.123 (protonated).

#### 2-[2-(4-Fluorophenyl)-6-methylimidazo­[1,2-*a*]­pyridin-3-yl]-*N*-(pyrrolidin-3-yl)­acetamide (**16**)

The core acid (**3**) was coupled with 1-Boc-3-aminopyrrolidine according to the general procedure. The product was then stirred overnight in 2 M HCl solution in diethyl ether. After this time, the mixture was flushed with compressed air and the solvent was evaporated. To prepare a free base, the compound was dissolved in methanol and stirred with potassium carbonate overnight. Then, potassium carbonate was filtered off and methanol evaporated to give compound (**16**) as a white solid, yield: 72%. ^1^H NMR (CD_3_OD, 500 MHz) δ 8.69 (br s, 1H), 7.80–7.95 (m, 2H), 7.74 (br dd, 2H, *J* = 5.2, 8.0 Hz), 7.40 (br t, 2H, *J* = 8.3 Hz), 4.47 (br s, 1H,), 4.21 (br s, 2H), 3.45–3.56 (br d, 2H, *J* = 6.2 Hz), 3.34–3.43 (br s, 2H), 2.55 (s, 3H), 2.29–2.41 (m, 1H), 2.03–2.19 (m, 1H) (NH protons not detected). ^13^C NMR (CD_3_OD, 126 MHz, single pulse quintuple) δ 170.1, 165.6, 140.0, 137.9, 134.9, 132.5 (2C), 129.7, 126.5, 124.3, 119.0, 118.0 (2C), 112.3, 51.3, 51.0, 45.8, 31.1, 30.9, 18.3. ^19^F NMR (CD_3_OD, 471 MHz) δ −111.21 (s, 1F). Formula: C_20_H_21_FN_4_O; MS (ESI^+^) *m*/*z* 353.183 (M+H^+^), calculated: 353.178 (protonated).

#### 2-(4-Fluorophenyl)-6-methyl-3-(2-oxo-2-(pyrrolidin-1-yl)-ethyl)­imidazo­[1,2-*a*]­pyridin-1-ium 3-carboxy-2,3-dihydroxy-propanoate (**17**)

The core acid (**3**) was coupled with pyrrolidine according to the general procedure. Compound (**17**) was obtained as a white solid, yield: 26%. Next, the obtained free base (1 equiv) was stirred with tartaric acid (1 equiv) in methanol for 1 h, and the solvent was evaporated to yield compound 9 as a salt with tartaric acid. Spectral data matched those reported previously:^41^ ^1^H NMR (CD_3_OD, 500 MHz) δ 8.17 (s, 1H), 7.60 (dd, 2H, *J* = 5.3, 8.9 Hz), 7.56 (d, 1H, *J* = 9.2 Hz), 7.40 (dd, 1H, *J* = 1.5, 9.2 Hz), 7.23 (t, 2H, *J* = 8.8 Hz), 4.45 (s, 2H), 4.12 (s, 2H), 3.57 (t, 2H, *J* = 6.8 Hz), 3.44 (t, 2H, *J* = 6.9 Hz), 2.38 (s, 3H), 2.0–2.0 (m, 2H), 1.90 (quin, 2H, *J* = 6.7 Hz). ^13^C NMR (CD_3_OD, 126 MHz) δ ppm 175.63, 168.77, 164.70 (d, *J* = 247.6 Hz), 143.53, 140.81, 132.46, 131.90 (d, *J* = 8.4 Hz) (2C), 129.40 (d, *J* = 3.3 Hz), 125.80, 124.55, 117.36, 117.10 (d, *J* = 22.0 Hz) (2C), 115.21, 73.82, 48.20, 47.55, 31.01, 27.17, 25.47, 18.29. ^19^F NMR (CD_3_OD, 471 MHz) δ −114.5 (s, 1F). Formula: C_20_H_20_FN_3_O; MS (ESI^+^) *m*/*z*: 338.275 (M + H^+^), calculated: 338.167 (protonated base).

### Radioligand Binding

The study was conducted in accordance with previously established protocols,^42^ and performed in duplicate. Rat brains were homogenized and prepared following the described methodologies. The assay was carried out directly on 96-well microplates containing 50 mM Tris-HCl buffer (pH 7.4) in a total volume of 300 μL. The reaction mixture consisted of 240 μL of brain tissue suspension, 30 μL of [^3^H]-muscimol, and 30 μL of the tested compound solutions (at concentrations ranging from 10^–10^ to 10^–5^ M). To determine nonspecific binding, 100 μM GABA was included in the mixture. The 96-well microplates were incubated at 0 °C for 10 min, followed by rapid filtration through FilterMate B glass fiber filters (PerkinElmer, USA) using a Harvester-96 MACH III FM (Tomtec, USA). The filters were gently dried in a microwave and sealed in plastic bags (PerkinElmer, USA). Each filter was then immersed in 10 mL of Ultima Gold MV liquid scintillation cocktail (PerkinElmer, USA). Radioactivity on the filters was subsequently measured using a MicroBeta TriLux 1450 scintillation counter (PerkinElmer, USA). *K*i values were calculated using the Cheng and Prusoff equation, and statistical analyses were conducted using GraphPad Prism (version 4.0, San Diego, CA, USA)

### Electrophysiological Studies

Electrophysiological experiments were conducted using the QPatch16X automated patch-clamp platform (Sophion Bioscience). HEK293 cells stably expressing the α1β2γ2 subunits of the human GABA-A receptor were cultured following standard protocols. On the day of the experiment, cells were detached from the culture flask using Detachin solution (VWR) and resuspended in a serum-free medium. The cell suspension was placed in a magnetic stirring tube onboard the automated electrophysiology system and allowed to recover at room temperature for 30 min. Subsequently, the cells were automatically transferred to the instrument’s built-in centrifuge, pelleted, and washed in extracellular Ringer’s solution. The cells were then applied to the pipetting wells of disposable 16-channel planar patch chip plates (QPlate 16X, with 10 patch-clamp holes per measurement site). Gigaseals were achieved using a combined suction/voltage protocol, and further suction was applied to establish the whole-cell configuration. GABA-A receptor chloride currents were recorded for 7 s after the addition of each compound. During the recordings, the holding potential was set to −90 mV, and experiments were conducted at room temperature. The extracellular solution contained (in mM): 2 CaCl_2_, 1 MgCl_2_, 10 HEPES, 4 KCl, 145 NaCl, and 10 glucose (pH 7.4, 300 mOsm). The intracellular solution consisted of (in mM): 140 CsF, 1 EGTA, 5 CsOH, 10 HEPES, and 20 NaCl (pH 7.2, 320 mOsm). The assay protocol was adjusted to determine the EC_50_ of the test compound by sequentially applying increasing concentrations of the compound (ranging from 0.001 μM to 1000 μM), coapplied with 10 μM GABA. The instrument software ensured a minimum interval of 60 s between compound additions. Typically, 5 μL of each ligand was added to the cells, followed 3 s later by a washout with extracellular solution (two cycles of 5 μL). In the allosteric modulator/antagonist mode, the cells were preincubated with the test compound or reference antagonist alone for at least 50 s prior to coapplication with the agonist. Data analysis was performed using QPatch Assay Software (v5.0, Sophion Bioscience), with results representing the mean of three independent experiments conducted on separate cells. The validation criteria for each experiment required that the current amplitude induced by GABA exceeded 500 pA and that the difference between the cell responses to the two GABA applications (AG1 and AG2) did not exceed 25%. The relative efficacy of each compound was calculated as the baseline-corrected ratio of the maximal current amplitudes recorded in the presence of the test compound and the reference agonist, expressed as (T1-ATG/AG1-ATG) or (T2-ATG/AG1-ATG).

### Permeability Assays

The A–B and B–A permeability assay using the MDR1-MDCKII cell line was conducted at Eurofins Discovery according to a well-established protocol.^75^

*PAMPA.* The precoated PAMPA Plate System Gentest, obtained from Corning (Tewksbury, MA, USA), was used for the permeability study. The system includes a 96-well receiver filter plate precoated with structured phospholipid layers and a matching donor microplate. Stock solutions of the test compounds and reference drugs were prepared in PBS buffer (pH 7.4) at a final concentration of 100 μM. Each compound solution (200 μL) was added to the donor wells and incubated at room temperature for 5 h. The quantity of molecules that penetrated from the donor wells to the acceptor wells through the phospholipid membrane was determined using UPLC-MS spectrometry (Waters ACQUITY TQD system with the TQ Detector, Waters, Milford, USA) with an internal standard. Permeability coefficients (P_e_, cm/s) were calculated according to the formula provided by the manufacturer.^76^

### Metabolic Stability

In Eppendorf tubes, potassium phosphate buffer (pH 7.4), a working solution of the test compound, and human liver microsomes (final concentration: 0.4 mg/mL, Sigma-Aldrich) were combined. The mixture was incubated at 37 °C with shaking for 15 min in a thermoblock. Following this, an NADPH regeneration system (Sigma-Aldrich) was added to the mixture, which was subsequently incubated with shaking at 37 °C for various time intervals (5, 15, 30, 60, 90, 120, 150, 180, 210, and 240 min). A control experiment was run in parallel, replacing the regeneration fraction with phosphate buffer. At the designated incubation times, an internal standard (levallorphan, Sigma-Aldrich) was added to each sample. To terminate the reaction, 70% perchloric acid (Sigma-Aldrich) was added, and the samples were centrifuged at 6000 rpm for 10 min at 4 °C. The resulting supernatants were collected and subjected to LC-MS/MS analysis. Each sample was analyzed in duplicate.

### In Vitro Studies

#### Cell Culture

The SH-SY5Y human neuroblastoma cell line was maintained according to established protocols. The cells, obtained from the American Type Culture Collection (ATCC), were cultured in Dulbecco’s Modified Eagle’s Medium (DMEM, high glucose; Thermo Fisher), supplemented with 10% fetal bovine serum (Thermo Fisher), 100 IU/ml penicillin (Merck), and 100 μg/mL streptomycin (Merck). They were grown in 75 cm^2^ flasks (Nunc) at 37 °C in a humidified atmosphere containing 5% CO_2_. For compound evaluation, SH-SY5Y cells were plated in 96-well plates (Falcon) at a density of 2 × 10^4^ cells per well. The cells were allowed to adhere and grow for 24 h in an incubator set to 37 °C and 5% CO_2_ before starting the experiments. Stock solutions of both test and reference compounds were prepared at a concentration of 10^–2^ M. A minimum of 1 mg of each compound was dissolved in dimethyl sulfoxide (DMSO). Serial dilutions were made in DMSO, then further diluted in PBS, mixed, and added to the cell culture medium.

#### Measurement of Cell Membrane Damage

Damage to the cell membrane was assessed using the bioluminescent ToxiLight bioassay (Lonza), a profound method for detecting cell toxicity. Following 24 h of treatment, 5 μL of the supernatant above the sediment was transferred to a 384-well plate (PerkinElmer). Subsequently, 20 μL of Adenylate Kinase Detection Reagent (AKDR) was added to each well. Luminescence was recorded after a 10 min incubation period using a POLARstar Omega plate reader (BMG Labtech). Results are presented as a percentage relative to the control sample (cells lysed), reflecting the proportion of dead cells compared to the control.

#### Cell Viability Assay

Cell viability was determined using the Presto Blue reagent (ThermoFisher) following the manufacturer’s instructions. After 24 h of incubation with the tested compound, Presto Blue was added to each microplate well in an amount equal to 10% of the remaining medium volume. The plates were then incubated at 37 °C for 15 min, and fluorescence (EX530; EM580 nm) was measured using a POLARstar Omega plate reader (BMG Labtech). The results, expressed as viability percentages, represent the proportion of live cells relative to the DMSO-treated control sample.

#### Intracellular Calcium Concentration

The intracellular Ca^2+^ levels were assessed using a homogeneous cell-based assay with the Fluo-4 Direct Calcium Assay Kit (Thermo Fisher). Following incubation with the tested compounds, the supernatant was discarded, and the cells were incubated with 1 μM Fluo-4/AM in FluoroBrite DMEM (Thermo Fisher) for 1 h at 37 °C. The results were expressed as a percentage relative to the control (DMSO). Fluorescence intensity was recorded using the ImageXpress Micro XLS system (Molecular Devices).

#### Oxidative Stress

Cellular reactive oxygen species (ROS) levels were determined using the CellROX Deep Red Reagent (Thermo Fisher). After completing the treatments, the medium was removed, and the cells were incubated in FluoroBrite DMEM (Thermo Fisher) containing 5 μM CellROX and 2 μg/mL Hoechst 33342. The results were expressed as a percentage relative to the control (DMSO). Fluorescence intensity was measured using the ImageXpress Micro XLS system (Molecular Devices).

### In Vivo Studies

#### Subjects

All behavioral procedures were conducted using drug-naive male Wistar rats (Charles River, Sulzfeld, Germany), (*n* = 8/per group). Animals were housed in groups of four per standard plastic cage under controlled environmental conditions (temperature: 22 ± 1 °C, humidity: 60%, and a 12:12 light-dark cycle with lights on at 07:00 a.m.). Animals were obtained from the breeder 2 weeks prior to the initiation of behavioral procedures, during which time they were weighed and handled multiple times. Animals had ad libitum access to tap water and standard laboratory chow (Labofeed H, WPIK, Kcynia, Poland). All animal treatments in this study adhered to the ethical standards outlined in Polish and European regulations (Directive 2010/63/EU). The procedures were reviewed and approved by the local ethics committee.

#### Open-Field Test, Amphetamine-Induced Hyperlocomotion and MK-801 Induced Hyperlocomotion Tests

All procedures were performed in a sound-attenuated experimental room between 9:00 a.m. and 3:00 p.m. Rats were transferred to the experimental room in their home cages 24 h prior to testing and allowed to habituate for 60 min. The following day, the open field test was conducted as described below. Locomotor activity was measured in black octagonal cages (80 cm diameter, 30 cm height) amid dim lighting and constant white noise (65 dB). Each animal was positioned at the midpoint of the open field and allowed to explore the entire area freely for 30 min. Locomotor activity (cm/30 min) was recorded and analyzed using an automated motion monitoring system (Videomot, TSE, Bad Homburg, Germany). Animals were habituated to the experimental room on the day before the open field test. Amphetamine (1 mg/kg) or MK-801 (0,3 mg/kg) were administered *i.p.* in a volume of 1.0 mL/kg, 15 min prior to the start of the open field test. Molecule 9 was suspended in 1.5% Tween and administered *i.p.* in a volume of 2.0 mL/kg, 30 min prior to the locomotor activity assessment. Forward locomotion (cm/30 min.) was investigated using a one-way analysis of variance (ANOVA) and the Newman-Keuls for individual post hoc comparisons. *p* values lower than 0.05 were considered significant. To analyze all data the Statistica 13.1 software (StatSoft, Tulsa, OK, USA) was used.

#### PPI Test

The PPI apparatus consisted of eight startle chambers (SR-LAB, San Diego Instruments, San Diego, CA, USA). Each contained a Plexiglas cylinder (8.9 cm in diameter × 20 cm in length) mounted on a Plexiglas frame and positioned within a sound-attenuated, ventilated enclosure. Acoustic stimuli and background noise were delivered through a loudspeaker positioned 24 cm above the animal. Startle responses, reflecting the movement of animals within the cylinder following an acoustic stimulus, were measured with a piezoelectric transducer positioned beneath the frame. SR-LAB software was employed to manage stimulus delivery and response recording The chamber light remained on, and the background white noise was maintained at 70 dB throughout the entire session. Animals were placed individually in the Plexiglas cylinder. Each session spanned 30 min and was initiated with a 5 min acclimatization period. The test session began with three initial startling stimuli (120 dB, 40 ms duration) delivered during the acclimatization period with an average intertrial interval (ITI) of 22.5 s (range: 15–30 s)

The preliminary stimuli were followed by 60 trials of varying intensities presented in random order, with a mean intertrial interval (ITI) of 22.5 s. Responses to the acoustic stimulus were captured over a 100 ms period beginning at stimulus onset. Startle amplitudes were calculated as the average response across 10 trials for each stimulation type. The PPI session comprised 10 background trials with a sham stimulus (70 dB, 40 ms), two sets of 10 prepulse trials (20 ms prepulse stimuli at either 84 or 90 dB), 10 pulse-only trials (120 dB, 40 ms startling stimulus), and two sets of 10 prepulse–pulse trials, each combining a 20 ms prepulse (84 or 90 dB) with the startling pulse. PPI magnitude was expressed as the percentage inhibition of startle amplitude relative to pulse-only (P) trials, using the formula: [(amplitude in P trials – amplitude in prepulse–pulse (PP-P) trials)/amplitude in P trials] × 100%. Startle responses to the initial three stimuli were excluded from all statistical analyses. MK801 was prepared in 0.9% NaCl and administered intraperitoneally (i.p.) at a dose of 0.6 mg/kg in a volume of 1 mL/kg, 15 min prior to the test. Tested compound was dissolved in 1.5% Tween and delivered i.p. at a volume of 2 mL/kg, 30 min before the experiment. Startle responses (in manufacturer’s arbitrary units), and magnitudes of PPI (%) were analyzed with the aid of a one-way analysis of variance (ANOVA). The Newman-Keuls test was used for individual post hoc comparisons. *p* values lower than 0.05 were considered significant. The Statistica 8.0 software (StatSoft, Tulsa, OK, USA) was used to analyze all data.

## Supplementary Material



## Notes

^1^H NMR, ^13^C NMR and LCMS/MS spectra of selected final compounds, biotransformation studies of the selected molecule (t_0,5_), cytotoxicity assay results, and PK properties of 17 (PDF)
