# Supplementary material for: Targeting GABAergic Hypofunction Associated with Schizophrenia: Identification of α1β2γ2GABA‑A Receptor Ligands with Neuroprotective and Antipsychotic Properties
Source: ACS Chem Neurosci. 2025 Jun 6;16(12):2277–94. doi: 10.1021/acschemneuro.5c00098 (PMC12186843; doi:10.1021/acschemneuro.5c00098)
Supplement: Supplementary file 1 [file cn5c00098_si_001.pdf]

Supporting Information

Targeting GABAergic Hypofunction Associated with Schizophrenia-Identification of α1GABA-A Receptor Ligands with Neuroprotective and Antipsychotic Properties

Barbara Mordyl[1],[‡] Katarzyna Szafrańska[1,2],[‡] Joanna Sniecikowska[1], Jakub Jonczyk[1], Bartłomiej Bieńko[1], Maria Mateos-Jimenez[1], Bartosz Wojdyła[1], Beata Gryzło, Monika Głuch-Lutwin[1], Agata Siwek[1], Tadeusz Karcz[1], Karolina Słoczyńska[1], Elżbieta Pękala[1], Alicja Zakrzewska-Sito[3], Pawel Mierzejewski[3], Marcin Kołaczkowski[1], Monika Marcinkowska[1*]

[1]Faculty of Pharmacy, Jagiellonian University Medical College, 9 Medyczna Street, 30-688 Krakow, Poland
[2]Doctoral School of Medical and Health Sciences, Jagiellonian University Medical College, Krakow 31-530, Poland
[3]Institute of Psychiatry and Neurology, 9 Sobieskiego Street, 02-957 Warsaw, Poland

Corresponding author: monika.marcinkowska@uj.edu.pl
‡These authors contributed equally.

Contents

1. Cytotoxicity studies for selected compounds **S2**

2. Metabolic stability studies of compound 6 in HLM, MLM and RLM **S3**

3. NMR and LCMS spectra of selected final compounds **S4**

4. PK parameters of compound 9 .... **S12**

1. Cytotoxicity studies for selected compounds

Table S1. The viability of the cells SH-SY5Y after treatment with tested compounds for 24 the results are expressed as a percentage of dead cells with luminescence ToxiLight Assay. The results are expressed as a per cent of control with whole lysis cells. N.C. not countable

	ZOLPIDEM			17			15			6		
**M**	**X [%]**	**SD**	**IC 50**	**X[%]**	**SD**	**IC 50**	**X[%]**	**SD**	**IC 50**	**X [%]**	**SD**	**IC 50**
1E-04	71	0.7	**N.C**	62	1.4	**N.C**	67	0.7	**N.C**	74	1.1	**N.C**
3E-05	90	2.8		92	0.7		96	2.5		87	3.5	
1E-05	92	1.4		100	1.4		108	1.4		93	2.8	
3E-06	96	1.4		99	2.1		105	1.5		105	0.7	
1E-06	98	2.1		103	4.9		110	2.1		109	1.8	
1E-07	100	0.7		102	2.8		107	0.9		97	4.9	
1E-08	101	0.7		97	4.9		108	1.7		108	1.4	
1E-09	100	1.4		101	1.4		101	1.4		101	0.7	

Table S2. The viability of the cells SH-SY5Y after treatment with tested compounds for 24 h the results are expressed as a percentage of live cells with fluorescence PrestoBlue Assay. The results are expressed as a per cent of vehicle (DMSO).
N.C. not countable

	ZOLPIDEM			17			15			6		
**M**	**X [%]**	**SD**	**IC 50**	**X [%]**	**SD**	**IC 50**	**X [%]**	**SD**	**IC 50**	**X [%]**	**SD**	**IC 50**
1E-04	18	0.5	**N.C**	15	0.7	**N.C**	14	0.2	**N.C**	24	0.4	**N.C**
3E-05	16	0.7		17	0.3		15	1.4		17	0.7	
1E-05	17	0.4		17	1.4		12	0.2		17	0.7	
3E-06	17	0.2		16	1.8		15	1.4		16	0.4	
1E-06	18	1.4		18	0.8		16	0.7		16	0.2	
1E-07	20	1.4		15	0.2		14	0.7		17	1.4	
1E-08	17	0.7		17	1.4		15	0.4		18	0.2	
1E-09	20	1.4		15	0.2		14	0.7		17	1.4	

2. Metabolic stability studies of compound 6 in HLM, MLM and RLM

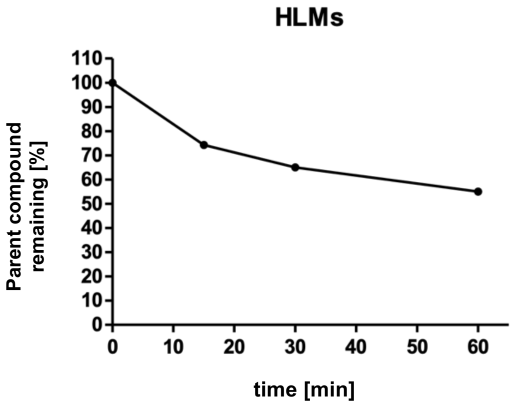

Fig. S1. *In vitro* biotransformation of compound **6** in human liver microsomes (HLMs). Percentage of the parent compound remaining over the incubation period

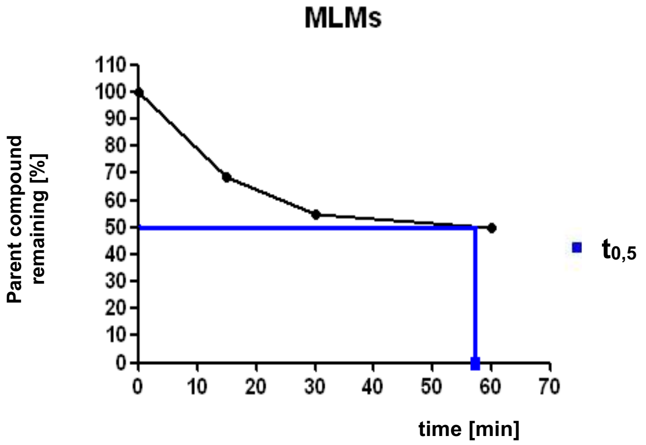

Fig. S2. *In vitro* biotransformation of compound **6** in mouse liver microsomes (MLMs). Percentage of parent compound remaining over the incubation period, with $t_{1/2}$ representing the half-life

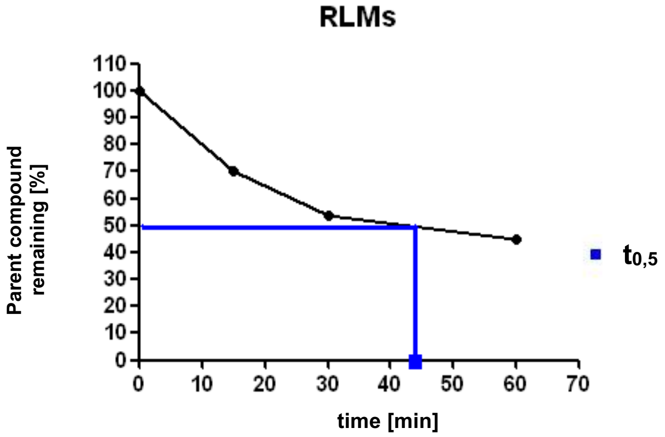

Fig. S3. *In vitro* biotransformation of compound 6 in rat liver microsomes (RLMs). Percentage of parent compound remaining as a function of incubation time, with $t_{1/2}$ indicating the half-life

3. NMR and LCMS spectra of selected final compounds

2-[2-(4-fluorophenyl)-6-methylimidazo[1,2-*a*]pyridin-3-yl]-1-(4-methylpiperazin-1-yl)ethan-1-one formate salt (4)

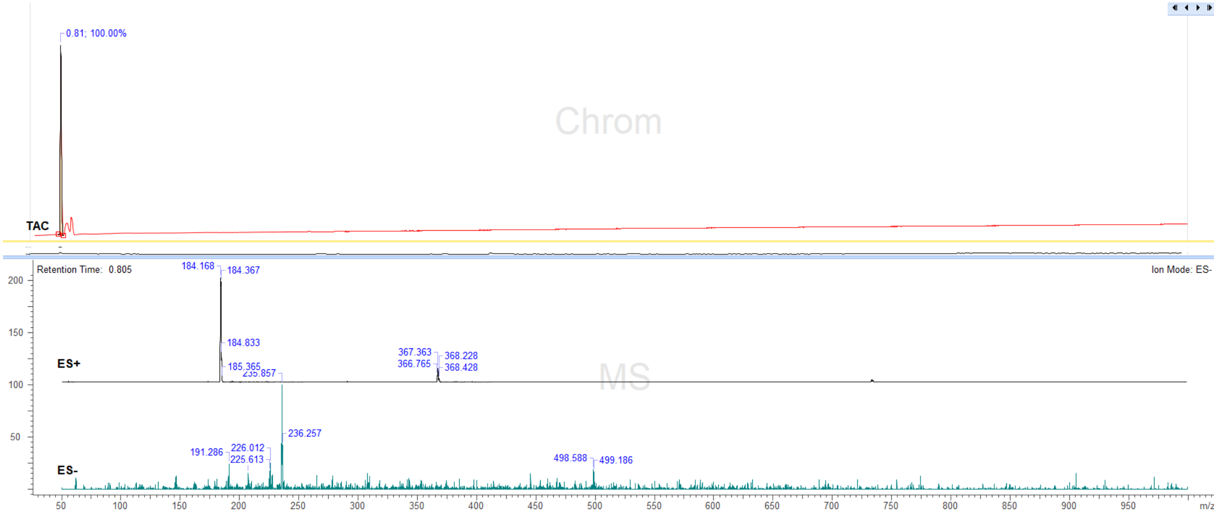

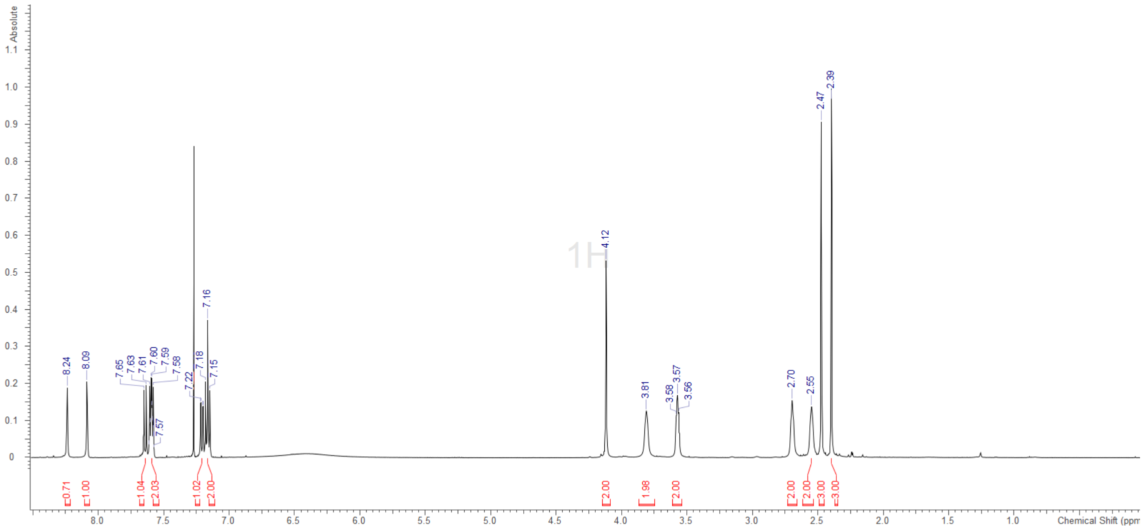

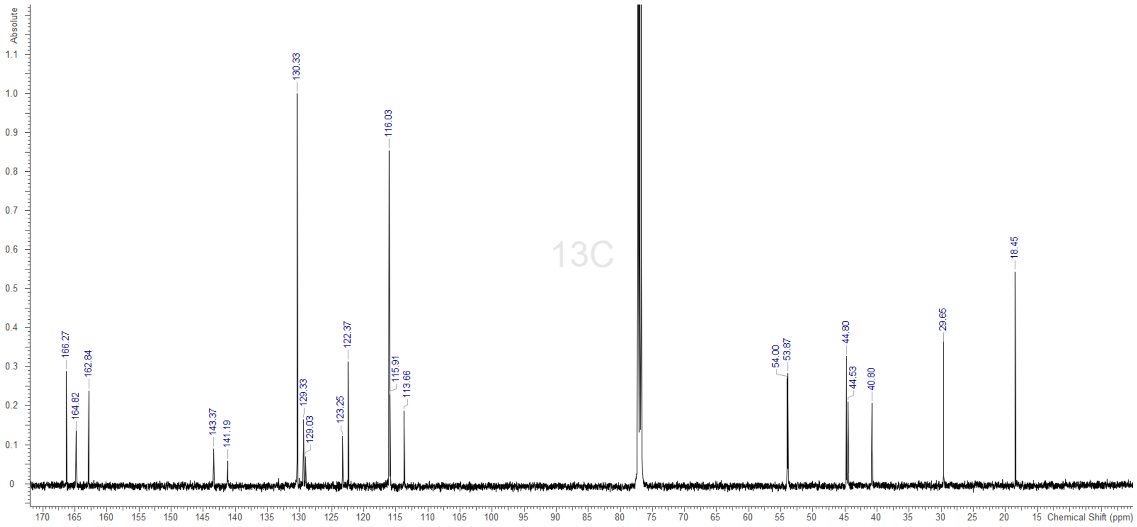

1-(3,5-dimethylpiperazin-1-yl)-2-[2-(4-fluorophenyl)-6-methylimidazo[1,2-*a*]pyridin-3-yl]ethan-1-one (6)

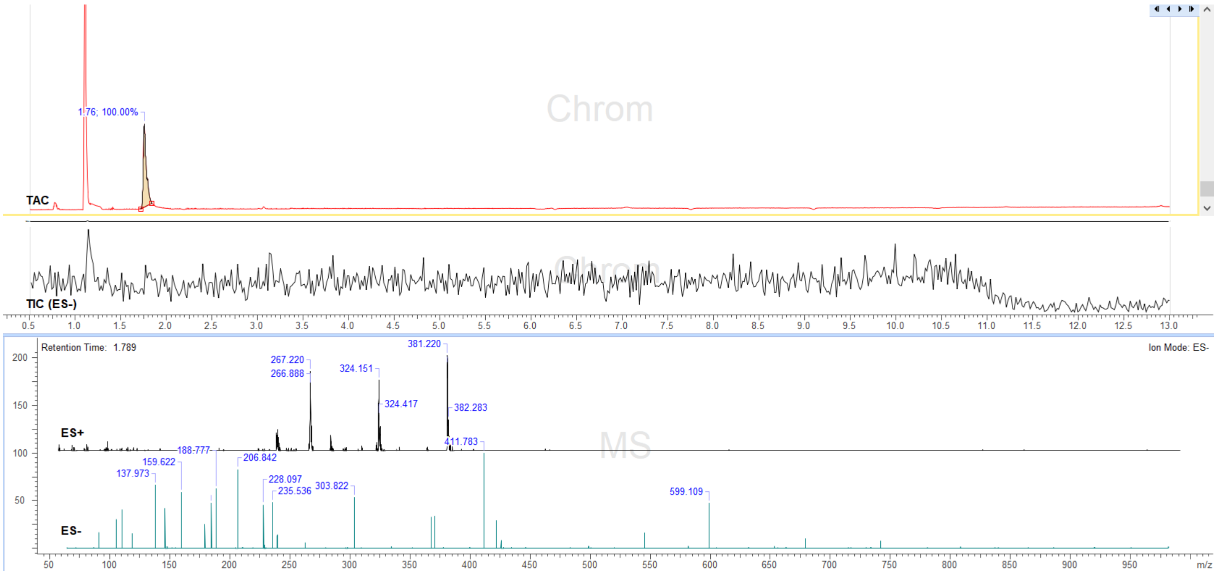

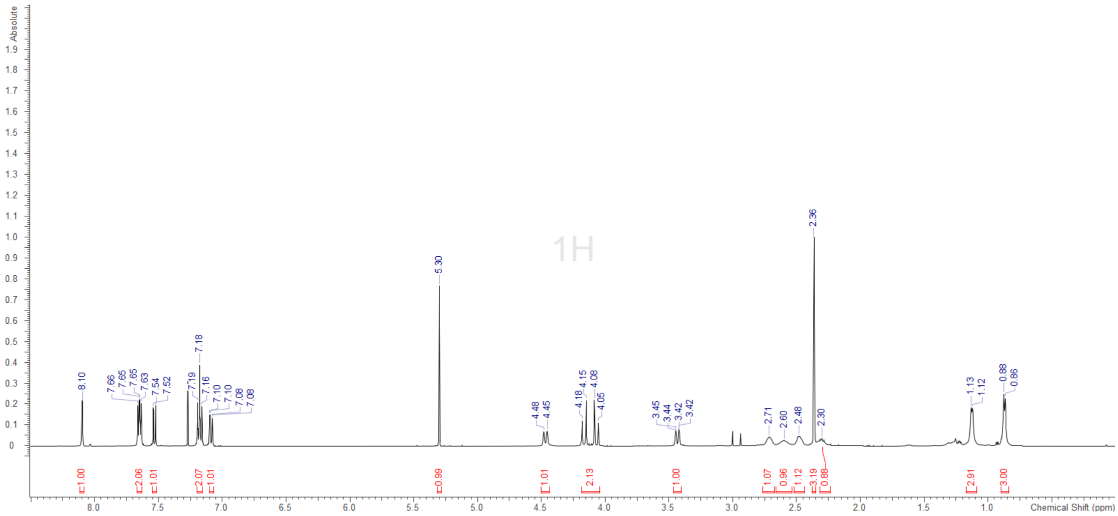

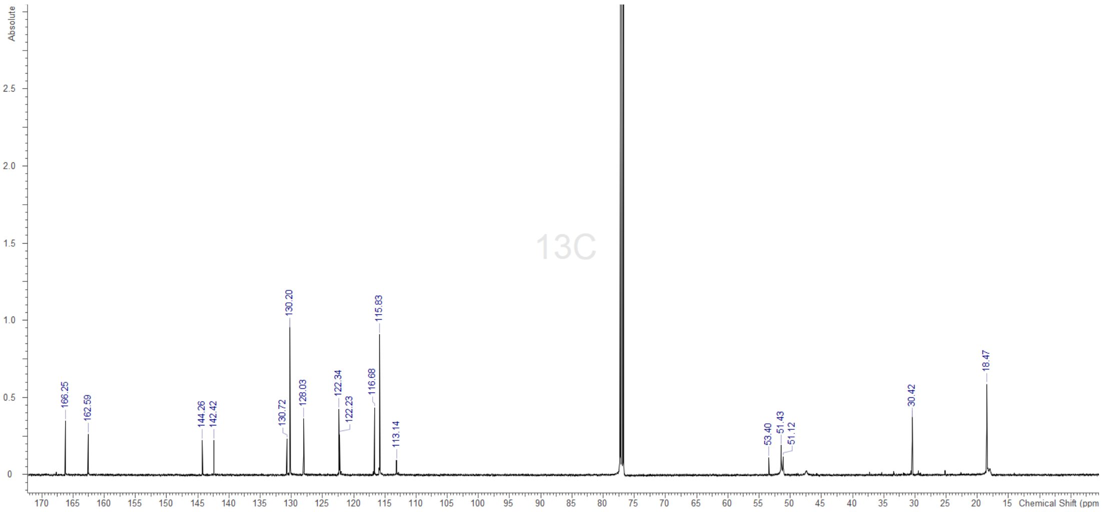

2-[2-(4-fluorophenyl)-6-methylimidazo[1,2-a]pyridin-3-yl]-1-(4-methylpiperidin-1-yl)ethan-1-one (7)

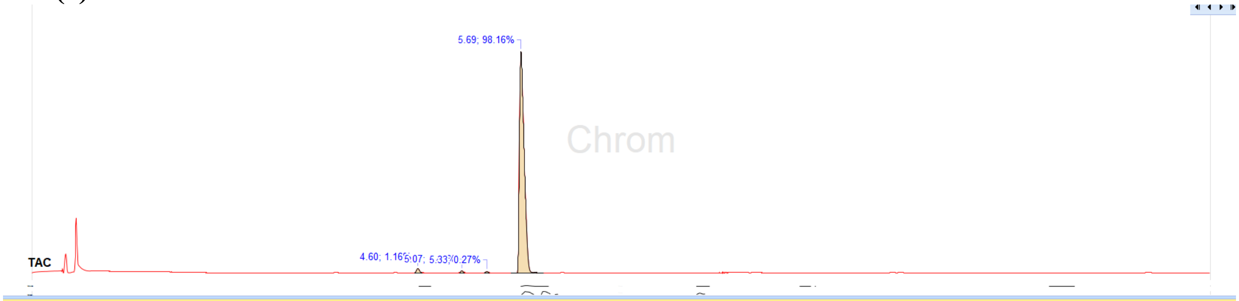

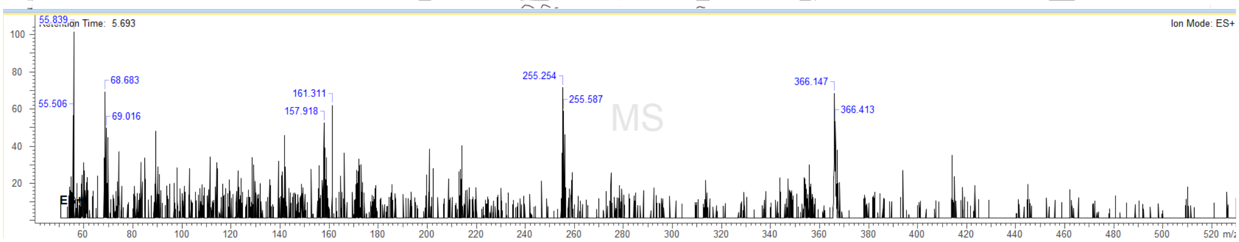

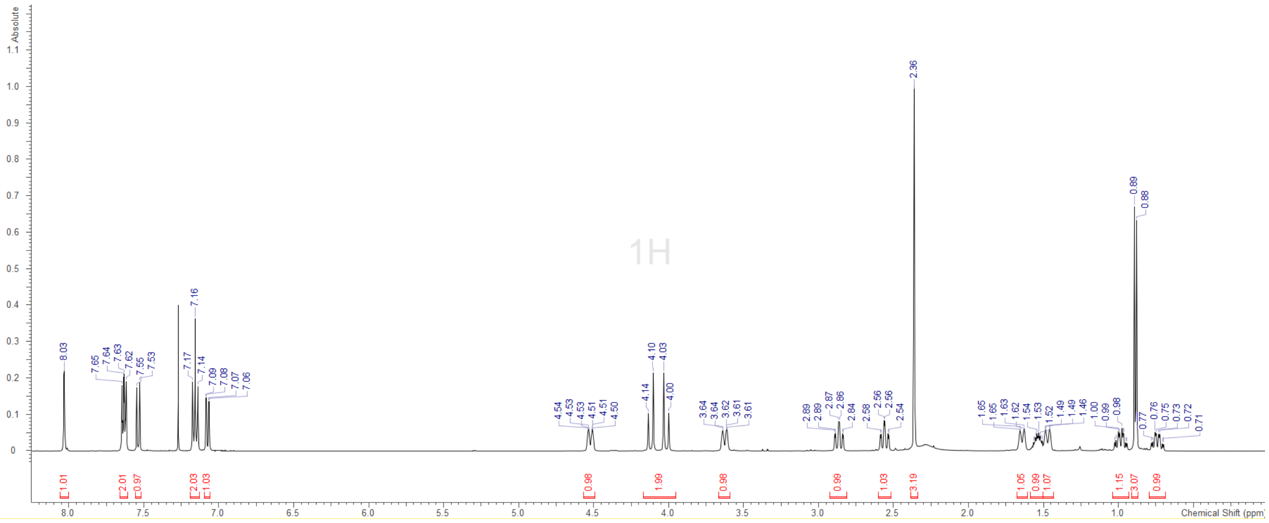

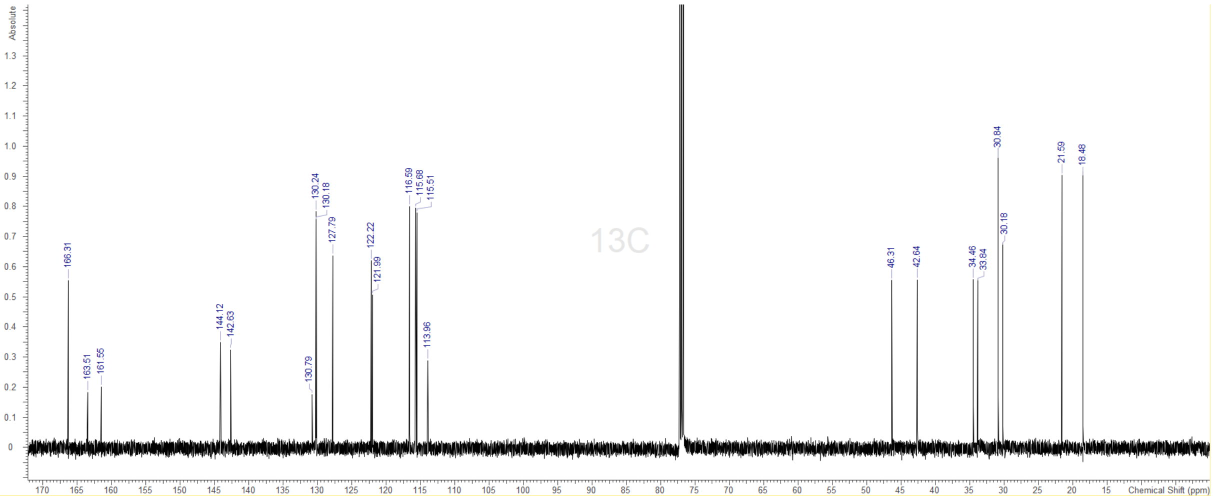

2-[2-(4-fluorophenyl)-6-methylimidazo[1,2-a]pyridin-3-yl]-1-[4-(2-methylpropyl)piperazin-1-yl]ethan-1-one (10)

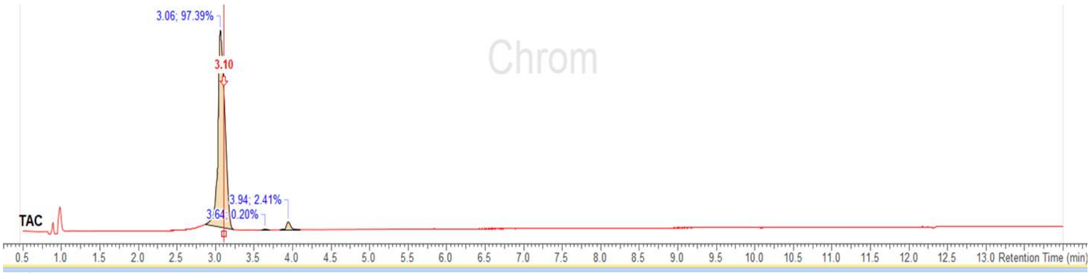

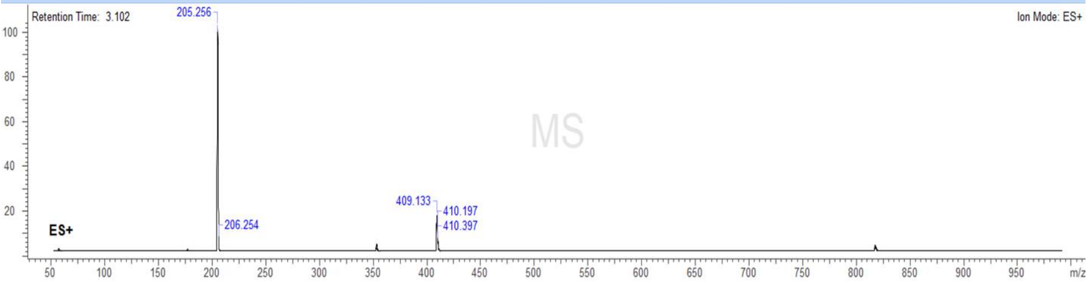

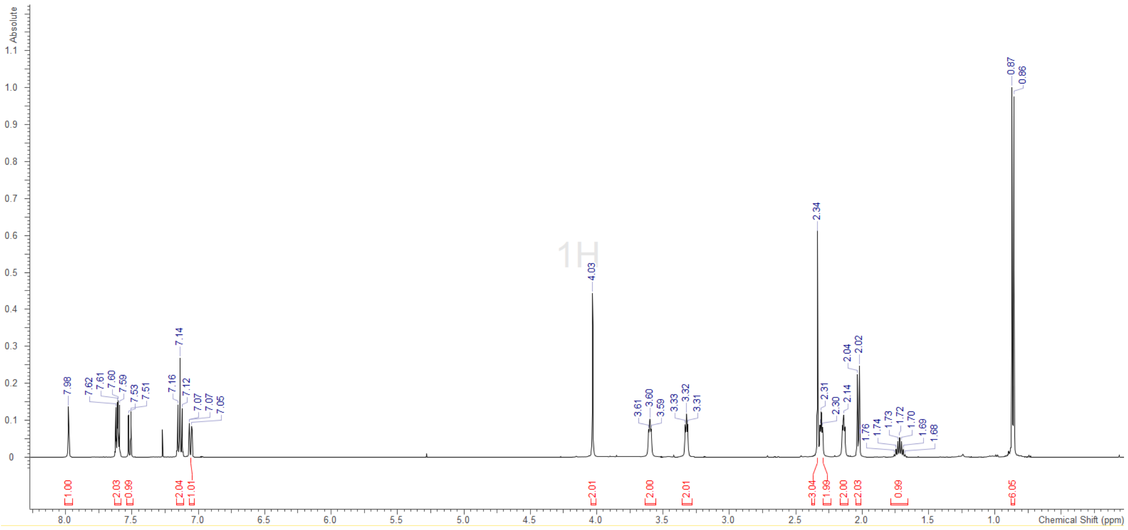

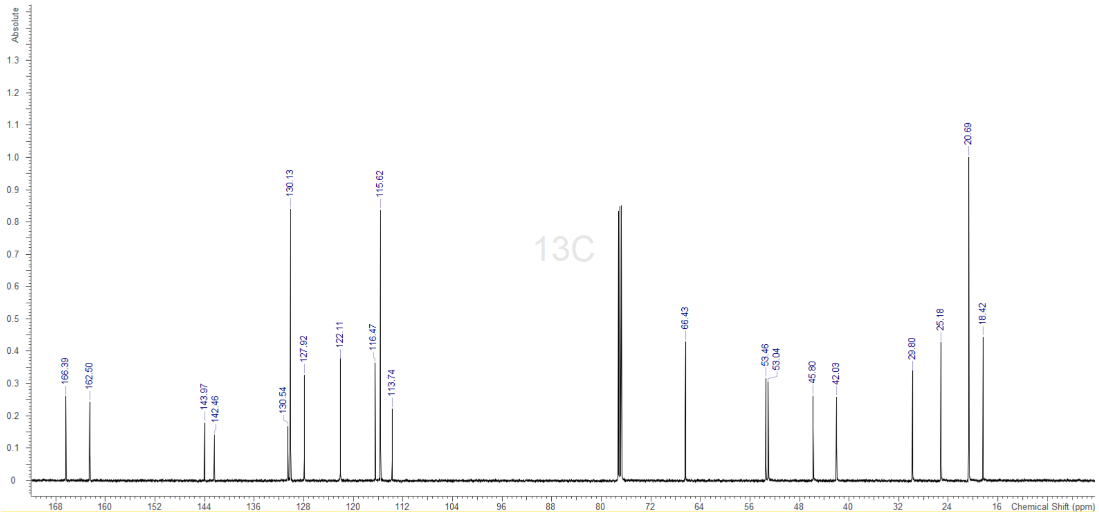

2-[2-(4-fluorophenyl)-6-methylimidazo[1,2-a]pyridin-3-yl]-1-(octahydro-1H-isoindol-2-yl)ethan-1-one (12)

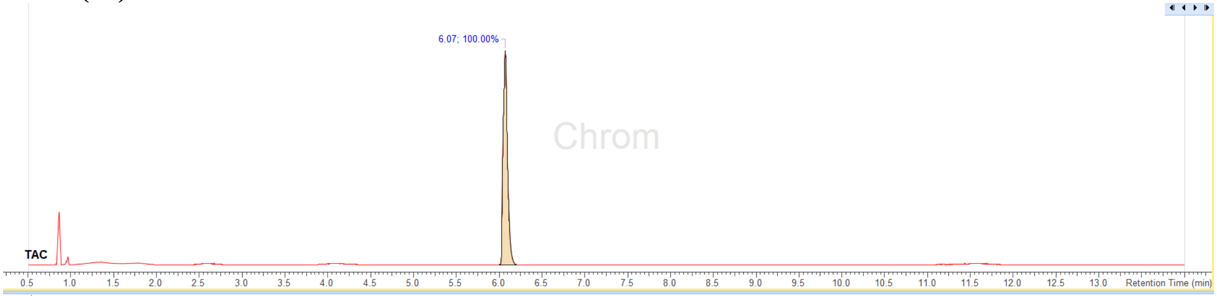

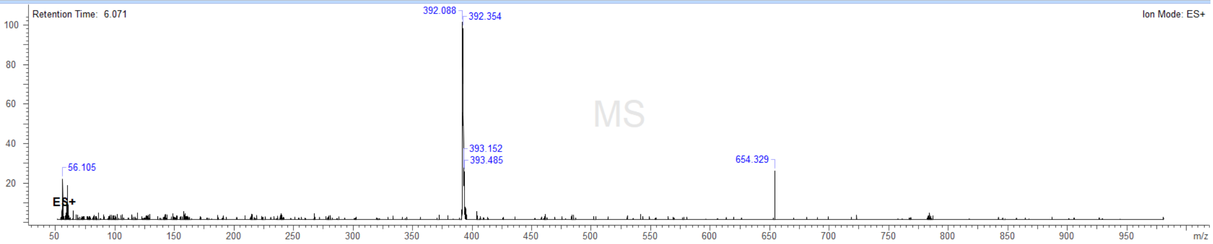

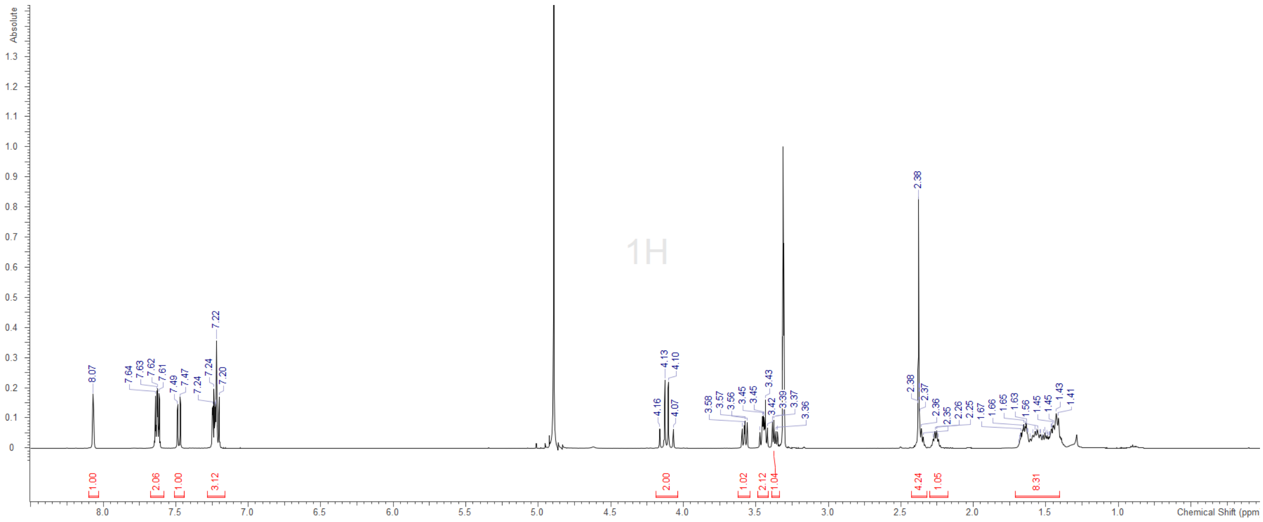

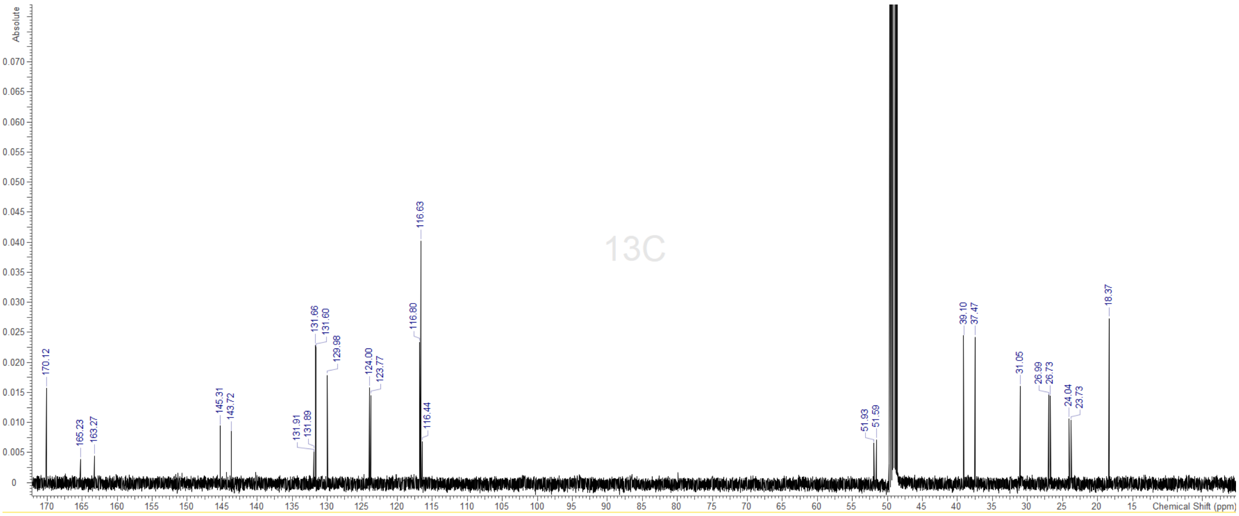

1-(2,2-dimethyl-1,3-thiazolidin-3-yl)-2-[2-(4-fluorophenyl)-6-methylimidazo[1,2-a]pyridin-3-yl]ethan-1-one (14)

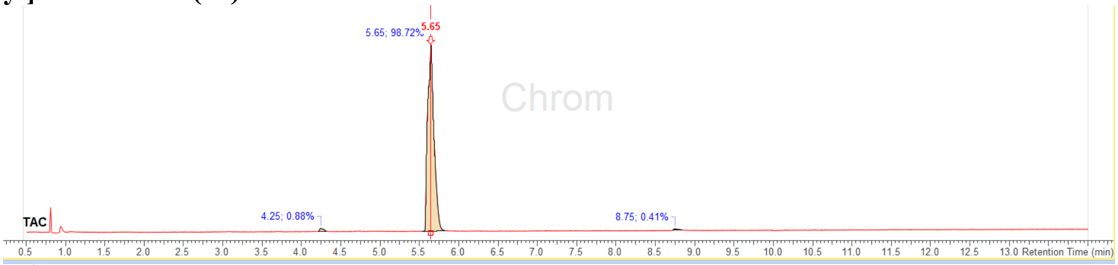

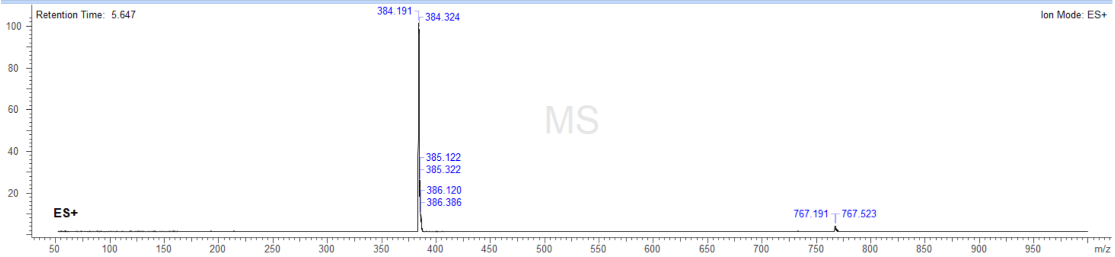

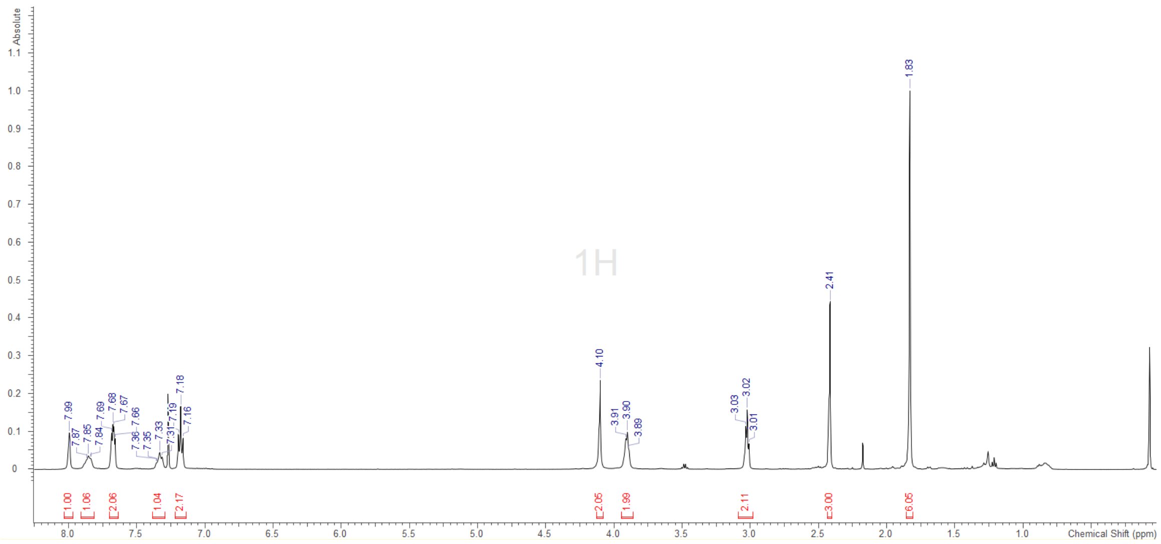

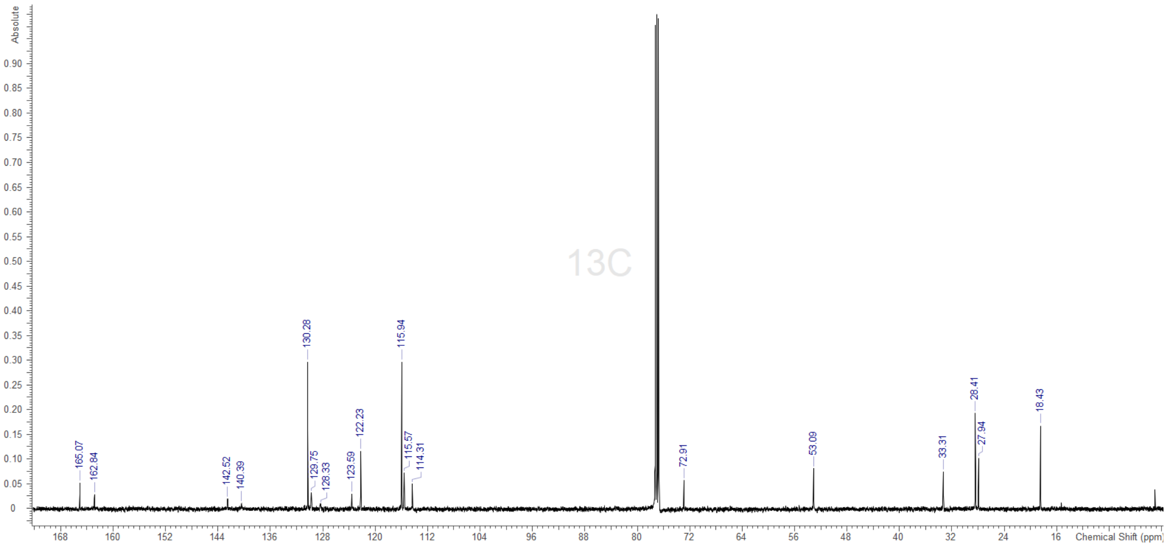

2-[2-(4-fluorophenyl)-6-methylimidazo[1,2-*a*]pyridin-3-yl]-1-(1,3-thiazolidin-3-yl)ethan-1-one (15)

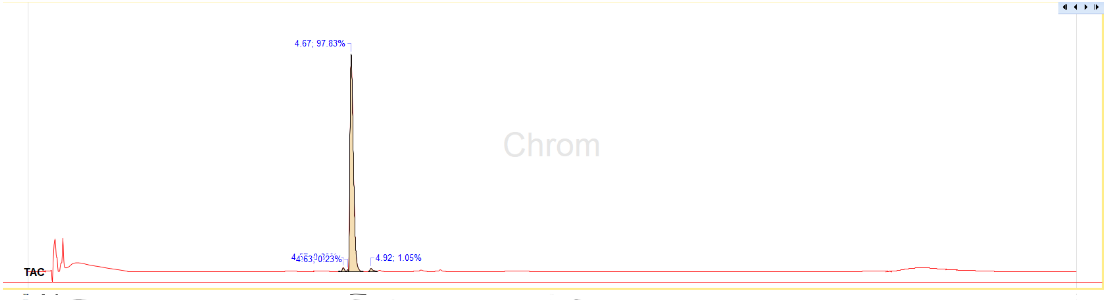

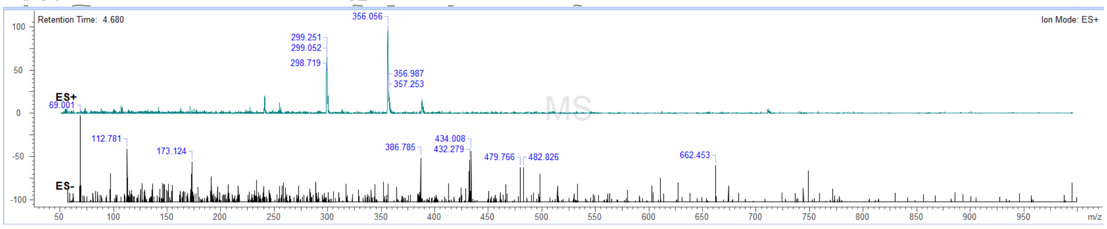

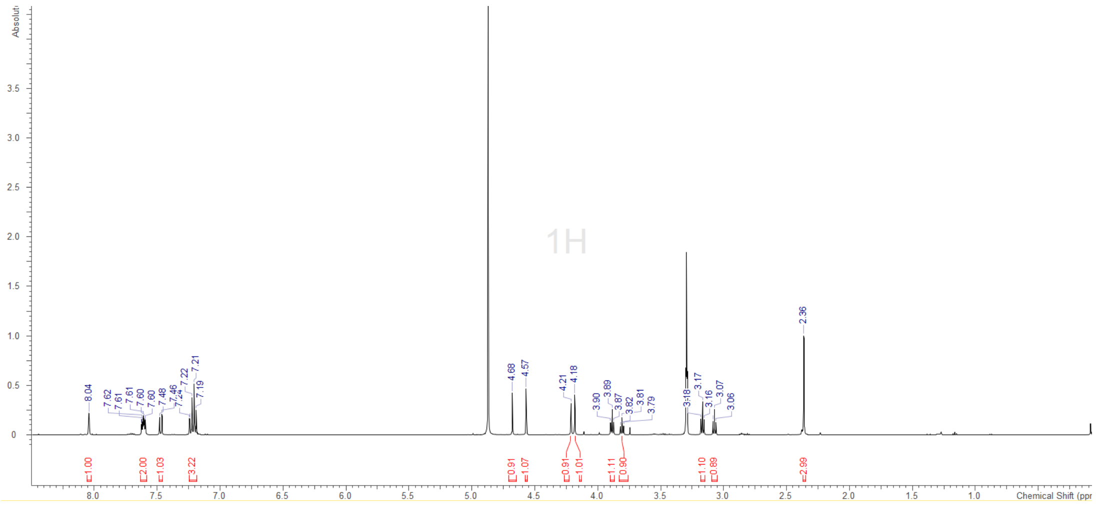

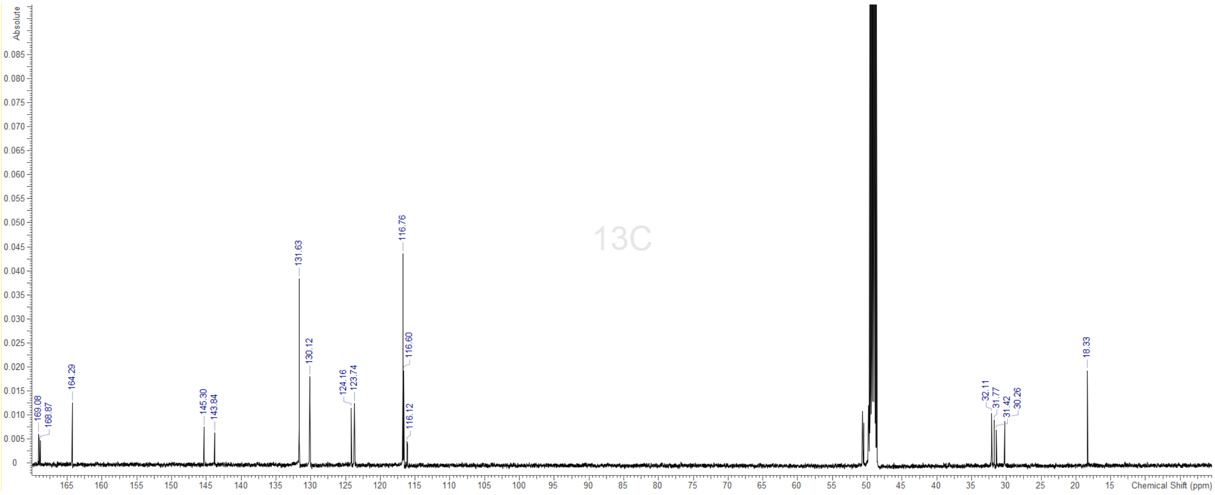

2-[2-(4-fluorophenyl)-6-methylimidazo[1,2-*a*]pyridin-3-yl]-*N*-(pyrrolidin-3-yl)acetamide (16)

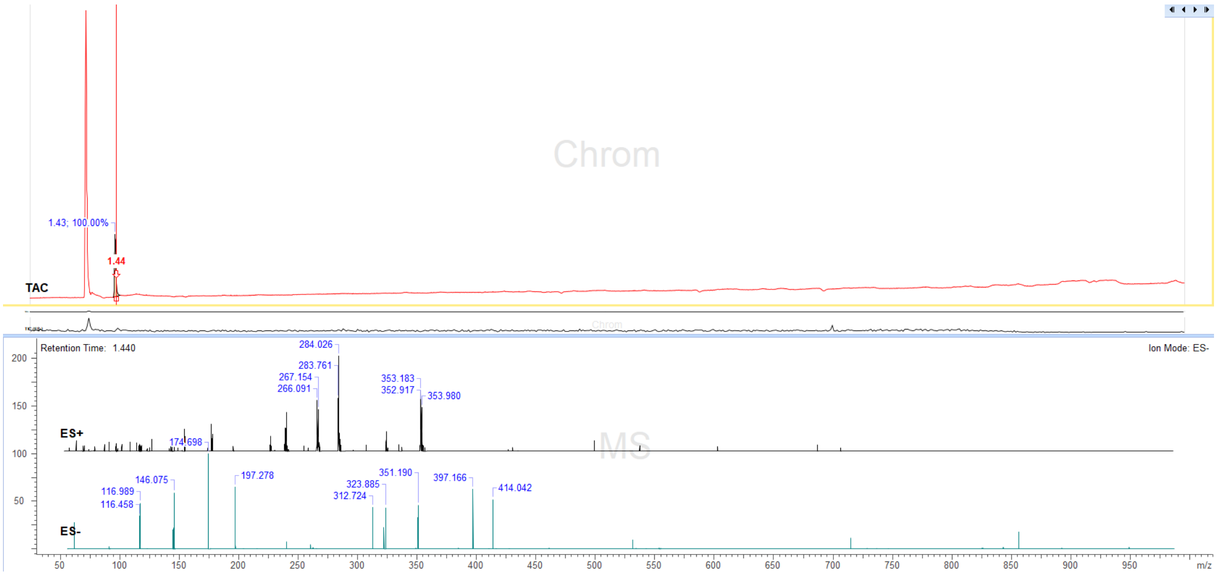

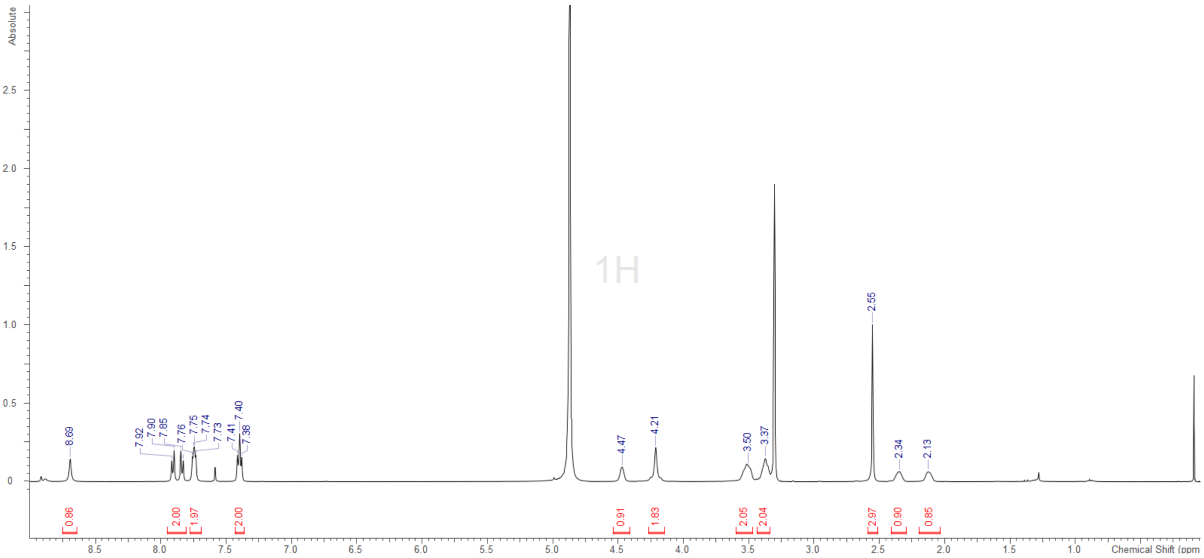

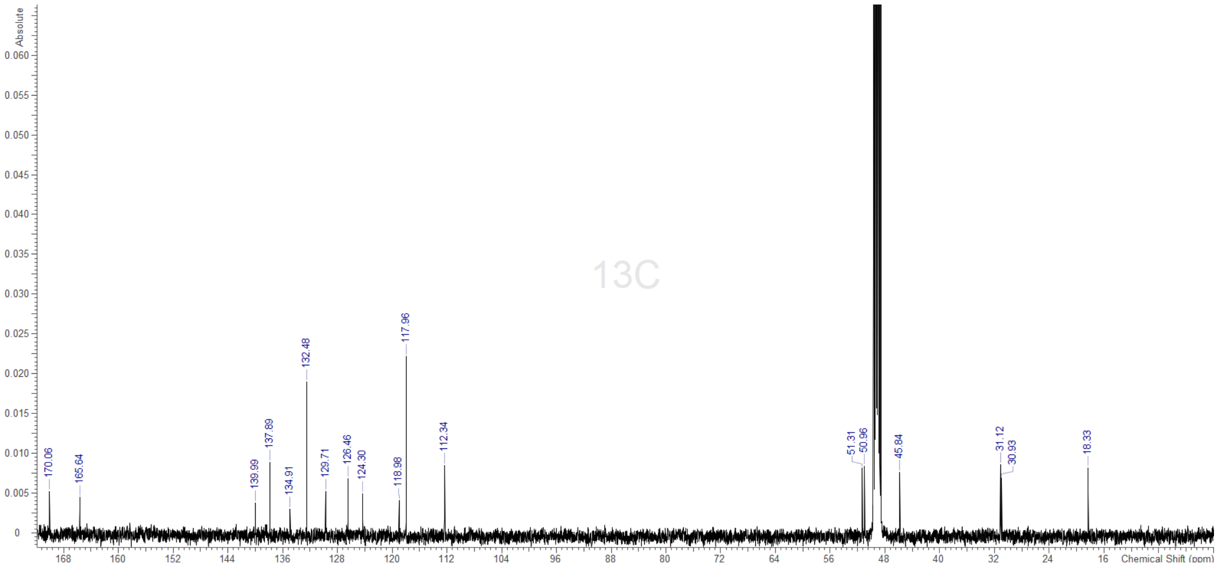

4. PK parameters of compound **17**

Table S3. Pharmacokinetic parameters for compound 17 determined after *i.p* administration at a dose of 3 mg/kg to rats (n=6)[1]

Parameters	Brain	Plasma
$AUC_{0\to t}$ [ng × min/g]	8229	128271 [ng × min/mL]
$t_{0.5}$ [min]	653.2	269.6
MRT [min]	102.5	50.9
C_{max} [ng/g]	219.5	4500
t_{max} [min]	5	5

AUC - area under the curve;
$t_{0.5}$ – terminal half life;
C_{max} - maximum plasma concentration; t_{max} - time to reach C_{max};
V_d – volume of distribution; Cl– clearance;
MRT – mean residence time
$t_{0,5}$ plasma= 269.6 min

[1]Mordyl, B. et al. Preferential Synaptic Type of GABA-A Receptor Ligands Enhancing Neuronal Survival and Facilitating Functional Recovery After Ischemic Stroke. *J. Med. Chem.* 2024, *67* (24), 21859–21889.
